# Supplementary material for: Multi-omics identify hallmark protein and lipid features of small extracellular vesicles circulating in human plasma
Source: Nat Cell Biol. 2025 Nov 28;27(12):2167–85. doi: 10.1038/s41556-025-01795-7 (PMC12717007; doi:10.1038/s41556-025-01795-7)
Supplement: Supplementary file 1 — Supplementary Figs. 1–22. [file 41556_2025_1795_MOESM1_ESM.pdf]

nature cell biology

Supplementary information https://doi.org/10.1038/s41556-025-01795-7

# Multi-omics identify hallmark protein and lipid features of small extracellular vesicles circulating in human plasma

In the format provided by the authors and unedited

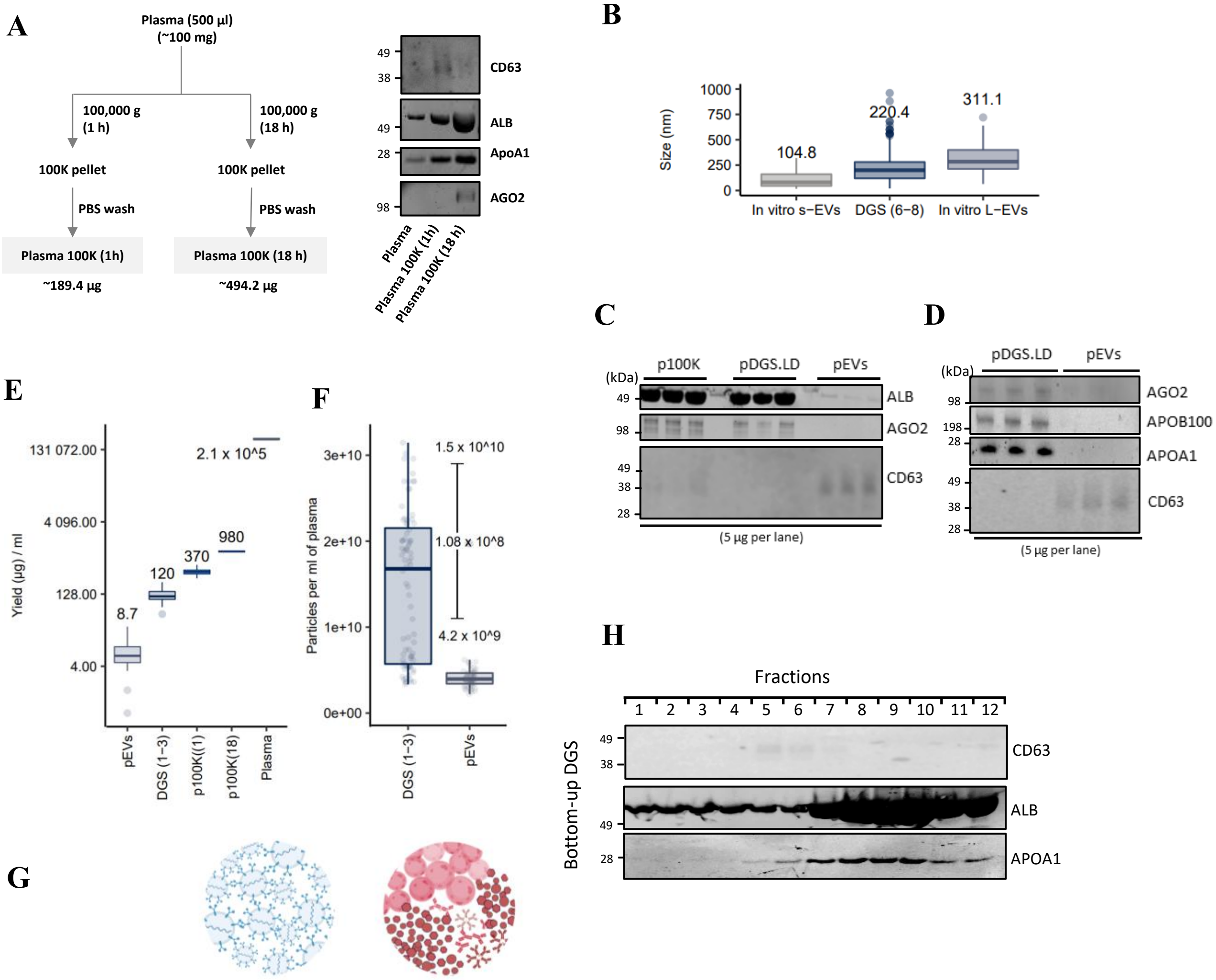


| | Plasma EVs | DGS (1-3) |
|---|---|---|
| Proteins | CD63, CD81 | ALB,APOs |
| Flotation density (g/ml) | 1.08-1.1 | 1.02-1.05 |
| Size (nm) | 30-250 | 30-250 |
| Particle morphology | Spherical | Spherical |
| Membrane-limited | Yes | No |
| Zeta potential | Negative | Negative |
| Enumeration | 4.2 x $10^9$ /ml | 1.5 x $10^{10}$ /ml |
| Protein yield | 8.7 µg/ml | 120 µg/ml |

**Supplementary Figure 1. Characterization of EVs from human plasma**. **A**. Ultracentrifugation of human plasma. The protein yields of plasma and isolated EV pellets are indicated. Western blot analysis of the crude EV pellets for indicated proteins. **B.** Cryo EM image-based size distribution of plasma EVs and previously reported sizes for small (s-EV) and large EVs (L-EVs). Boxplots show the median (centre line), 25th–75th percentiles (box), minima and maxima within 1.5× interquartile range (whiskers), and outliers beyond. **C-D.** Western blot analysis of indicated DGS fractions (p100K, ultracentrifugation; pDGS.LD, DGS fractions (1-3); pEVs, DGS fractions 6-8) from 6 different pooled plasma samples (n=6 independent plasma samples, in two independent experiments). Protein load per lane as indicated. **E.** Protein yield (µg) of pEVs per ml of plasma. **F.** Nanoparticle tracking analysis based total particle counts in DGS fractions (1-3) and DGS fractions (6-8)/pEVs per ml of plasma (n=7). Boxplots in **E-F** show the median (centre line), 25th–75th percentiles (box), minima and maxima within 1.5× interquartile range (whiskers). **G.** Comparative summary of biophysical characterization of pEVs and NonEVs. **H.** Bottom-up density gradient separation (DGS) of human plasma (0.5 ml). Western blot analysis of twelve DGS fractions (vol:vol matched) of human plasma with antibodies against indicated proteins.

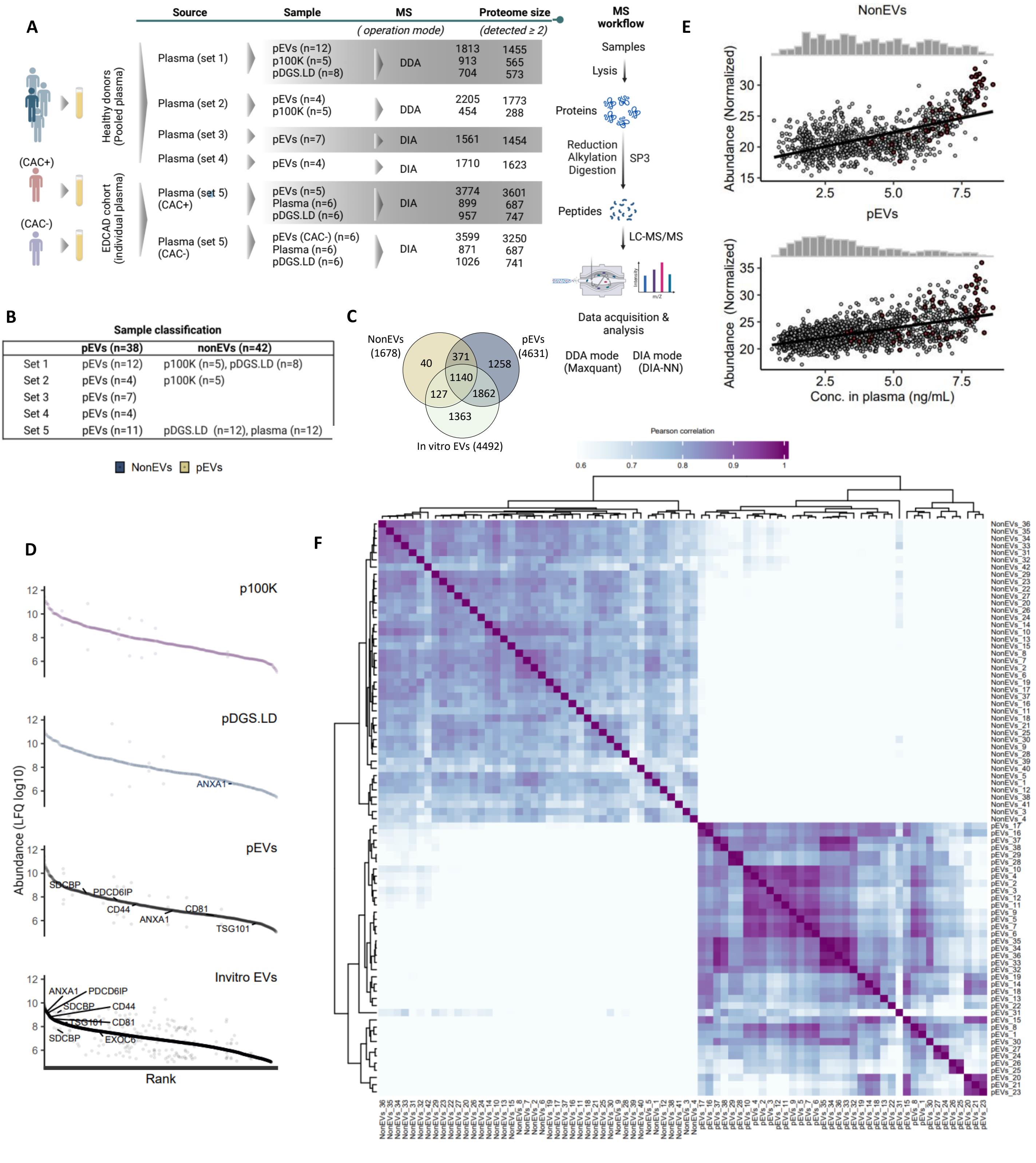


**Supplementary Figure 2. Construction and characterization of circulating EV proteome landscape**. **A.** Workflow depicting sample details and MS-acquisition modes for constructing proteome landscape of plasma EVs (pEVs). Different experimental groups are indicated as Set, totalling six sets. The figure was generated using BioRender. **B**. Classification of different proteomes (from sets 1-5) into pEVs or nonEV groups. Bar plot depicts number of proteins identified within each proteome. Box plot represents normalised protein abundance (log10 intensity). **C.** Venn diagram of proteins identified indicated proteomes. **D.** Protein ranked by their abundance within indicated proteomes in Set 1. Proteins typical reported as EVs proteins are highlighted. **E.** Correlation of proteins in plasma, pDGS.LD and pEVs proteomes (median protein intensities of replicates) to the published concentration of the same proteins[49]. The black lines are linear regression models (the grey shaded regions represent 95% confidence interval). **F.** Heatmap of Pearson correlation matrix of protein abundance between proteomes.

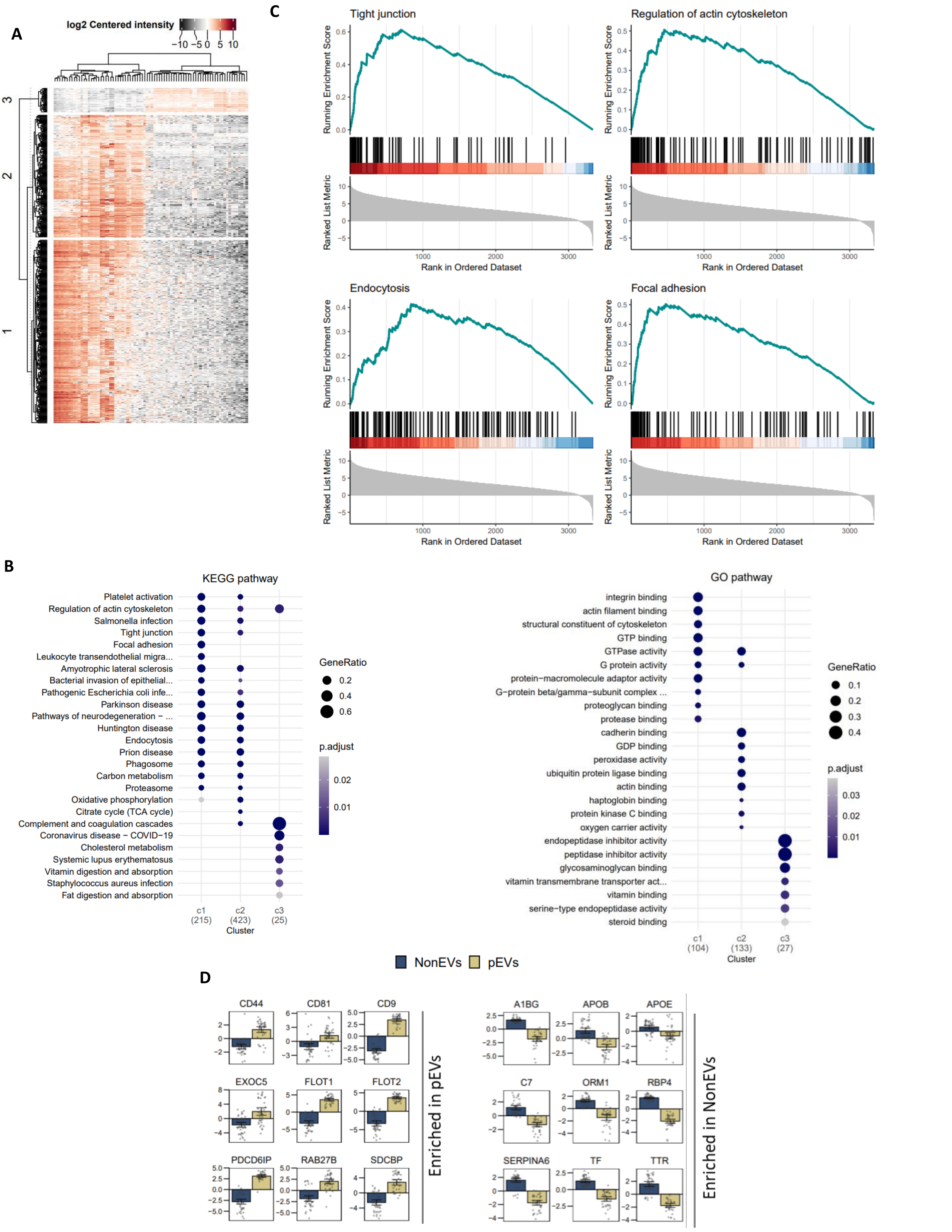


**Supplementary Figure 3. Classical hallmark features of EVs preserved in circulating EV proteome**. **A.** Heatmap of differentially abundant proteins ($p<0.05$, log2 FC $>1.5$) with k-means clustering. **B.** KEGG pathways and Gene Ontology pathways enriched in each cluster (Benjamini–Hochberg corrected p.adjust $< 0.05$). **C.** Leading edge analysis indicated for selected GSEA-KEGG pathways enriched in pEVs vs NonEVs. **D.** Bar plots showing relative abundance (log2 centred MS-based intensities) of differentially abundant EV and abundant plasma proteins; error bars denote the standard error of the mean.

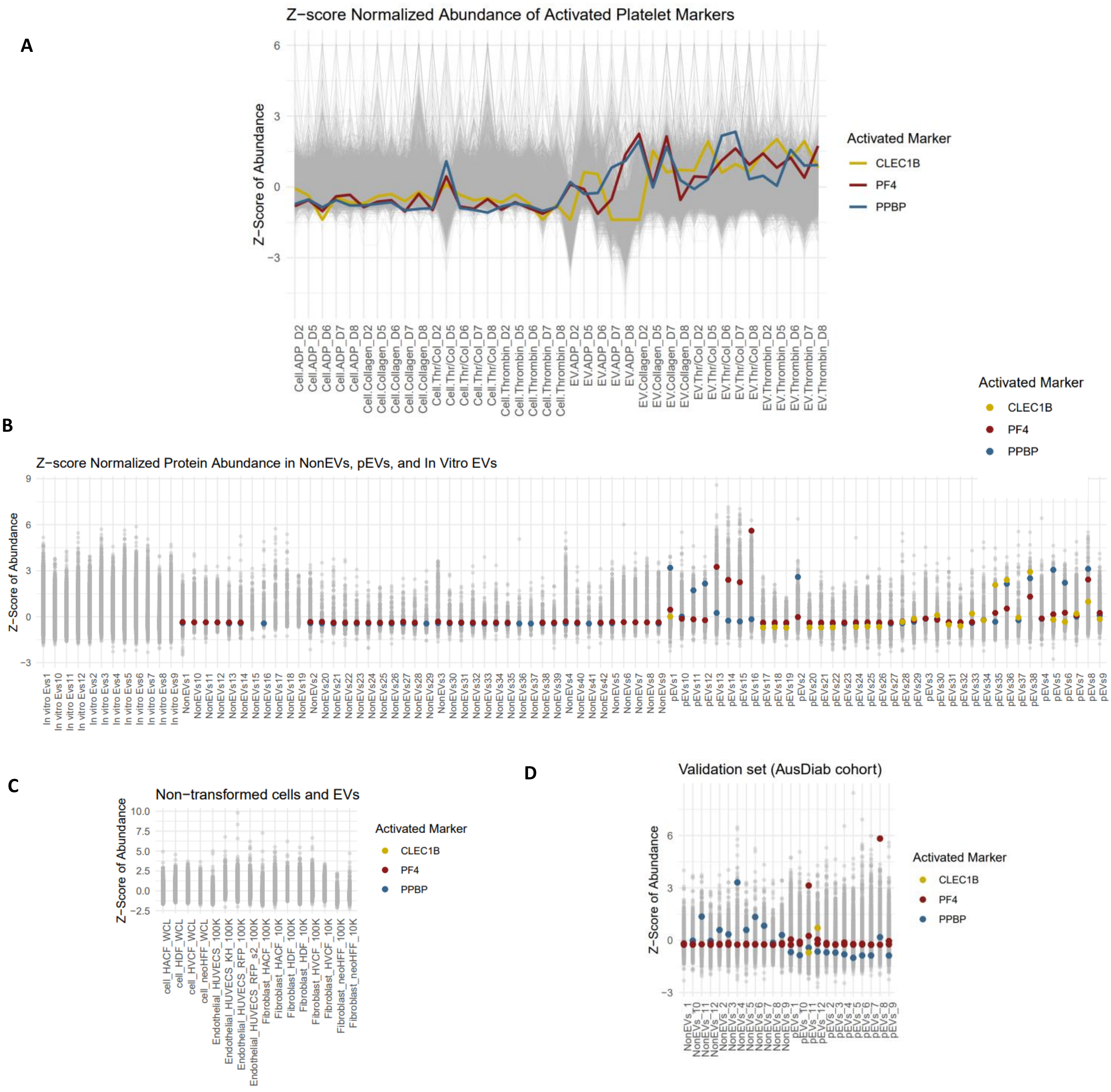


**Supplementary Figure 4. Quantification of platelet EV proteins in circulating EV proteome**. **A.** Detection of platelet proteins (CLEC1B, PF4, PPBP) in platelet EVs in our previous report[53]. **B.** Detection of platelet EV proteins (CLEC1B, PF4, PPBP) in in vitro EV, NonEV or pEV proteomes. **C.** Detection of platelet EV proteins (CLEC1B, PF4, PPBP) in non-transformed cell-derived EVs. **D.** Detection of platelet EV proteins (CLEC1B, PF4, PPBP) in pEVs and NonEV proteomes from AusDiab Validation set.

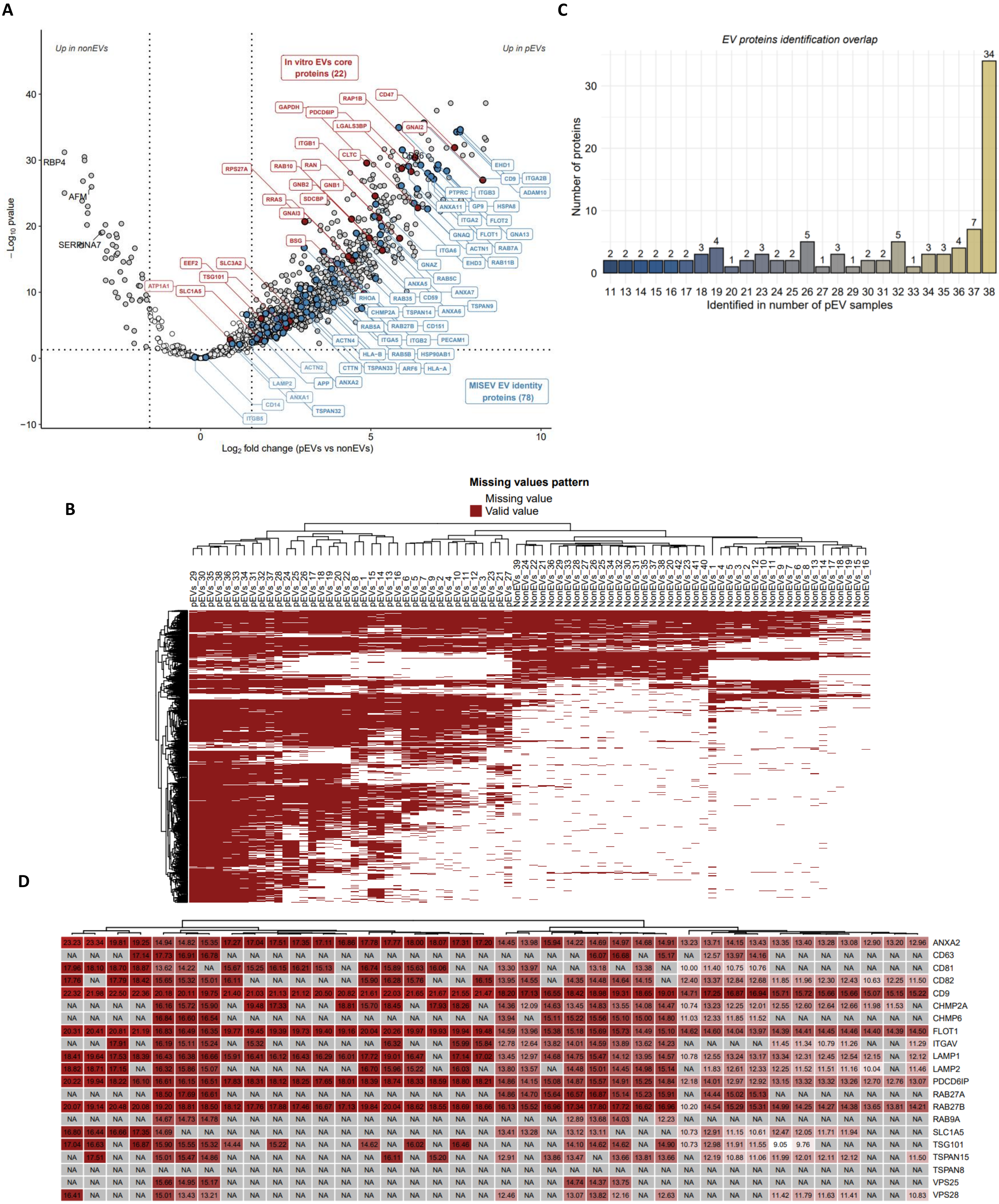


**Supplementary Figure 5**. **Mapping EV markers in circulating EV proteome. A.** Pair-wise comparison of differentially abundant proteins ($p<0.05$, FC > 1.5) depicted as a volcano plot where 22 core proteins established for in vitro EVs[30] and ninety-four EV marker proteins recommended by MISEV[36] guidelines are depicted. **B**. Heatmap showing quantification of current EV marker proteins in pEVs. **C**. Occurrence analysis for current EV marker proteins in 38 pEV proteomes. **D**. Heatmap of selected EV proteins depicting their identification and abundance in 38 pEV proteomes.

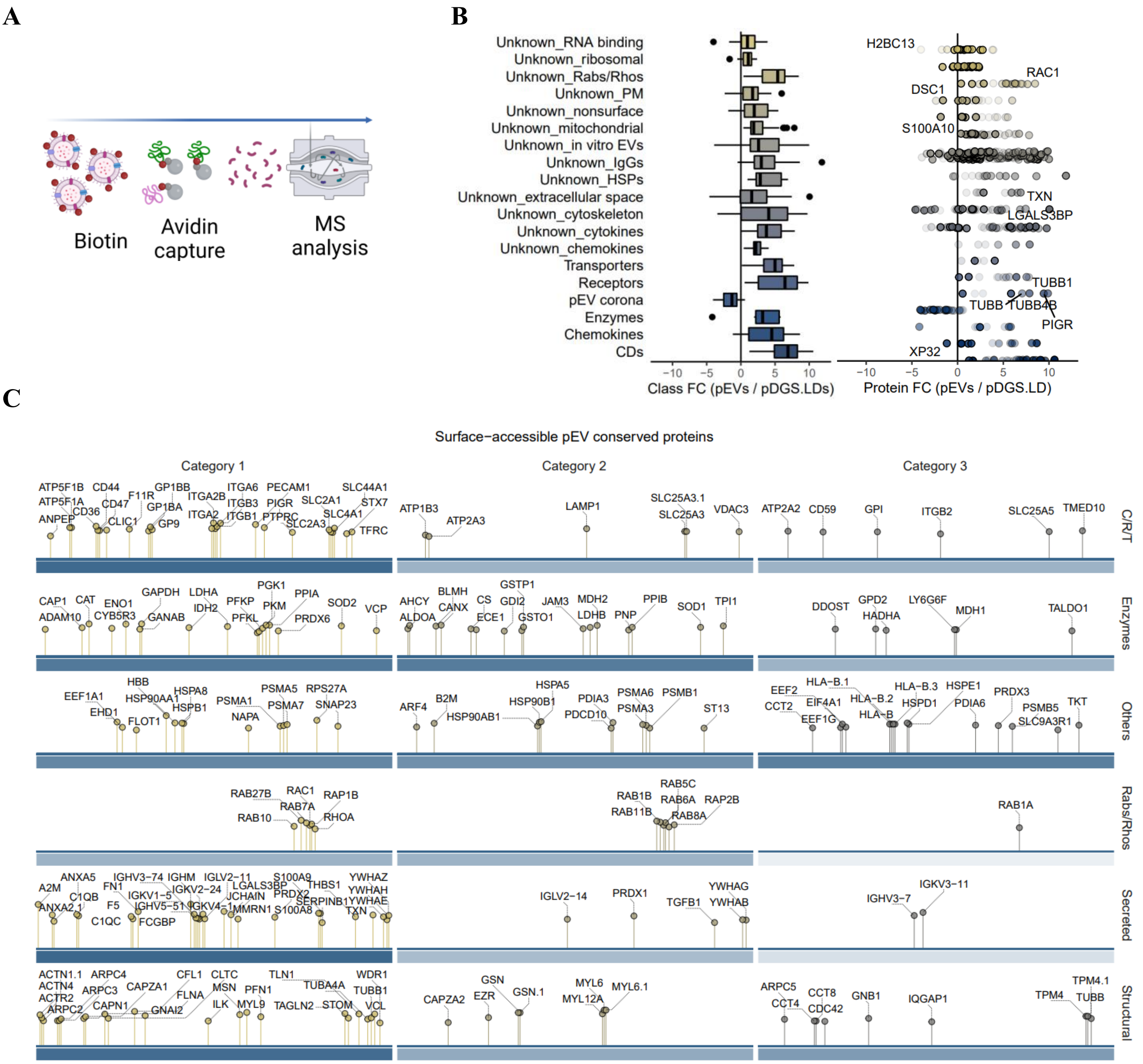


**Supplementary Figure 6**. **Surfaceome of circulating EVs in human plasma. A.** Workflow for capture and identification of EV surface proteins. **B.** Categorization of pEV surface proteins and their relative abundance in pEVs vs pDGS.LD proteomes. **C.** Surface-accessible Category 1-3 proteins of pEVs categorised based on their molecular/functional annotation. C/R/T represents cluster of differentiation (CDs), receptors and transporters.

**A**

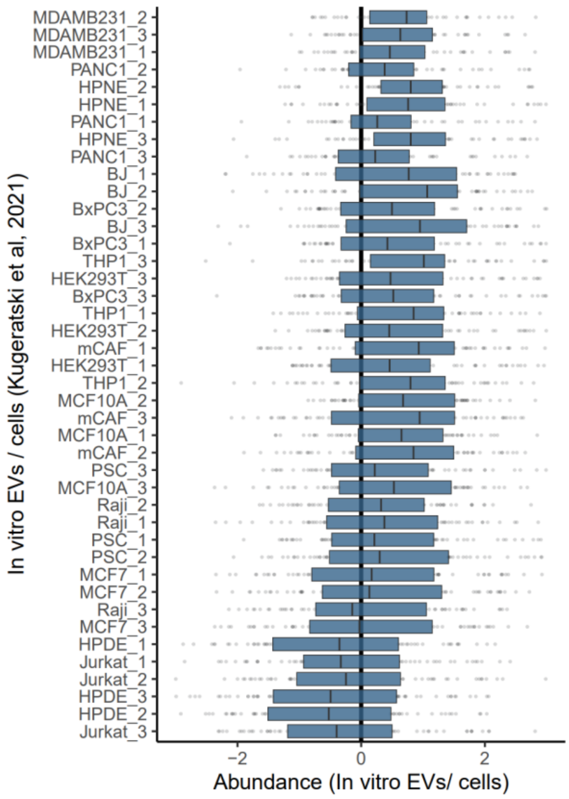


**B**

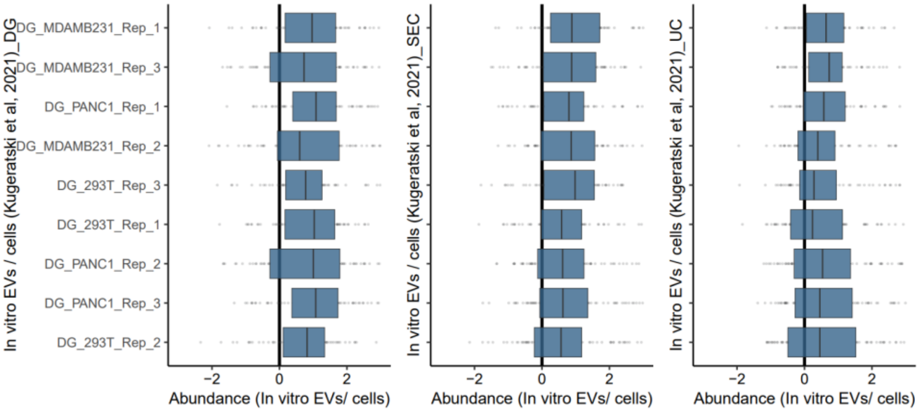


**Supplementary Figure 7. Conservation of circulating EV protein features in cell culture-derived EVs published proteomes. A.** Relative abundance of EV protein features in sEVs vs their parental cell proteome as reported[30], whereby EVs were isolated from culture media using ultracentrifugation (UC). **B.** Relative abundance of EV protein features in sEVs vs their parental cell proteome as reported[30], where EVs were isolated using density gradient separation (DG), size-exclusion chromatography (SEC), and UC. Boxplots show the median, 25th–75th percentiles (box).

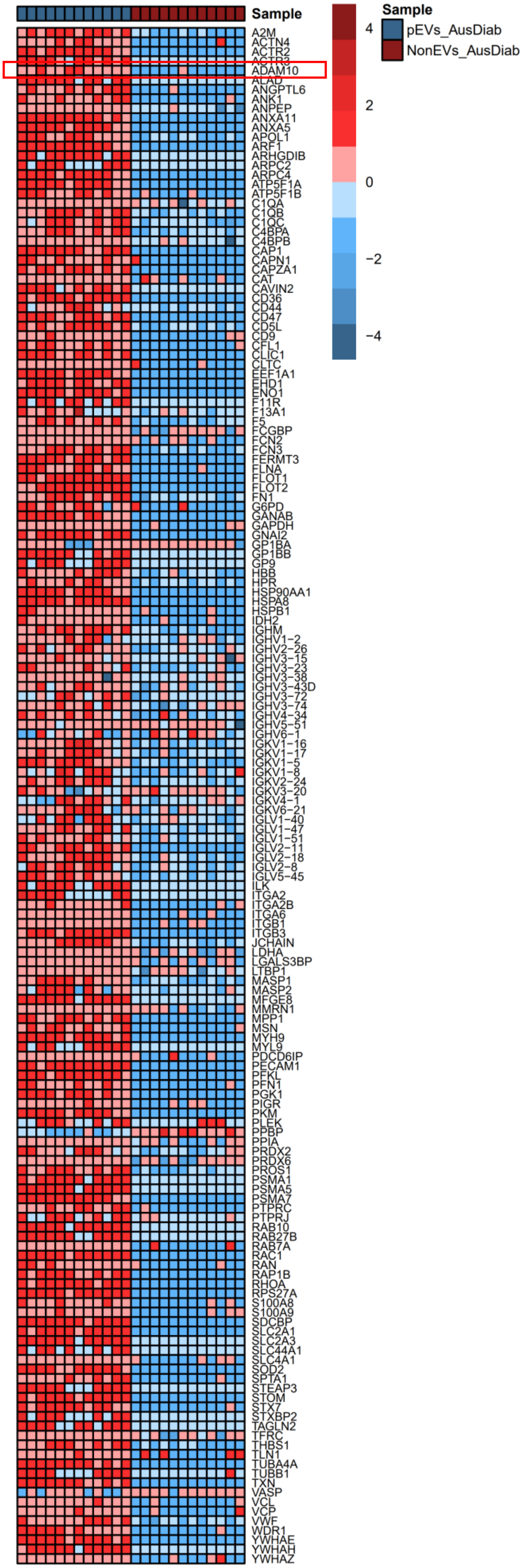


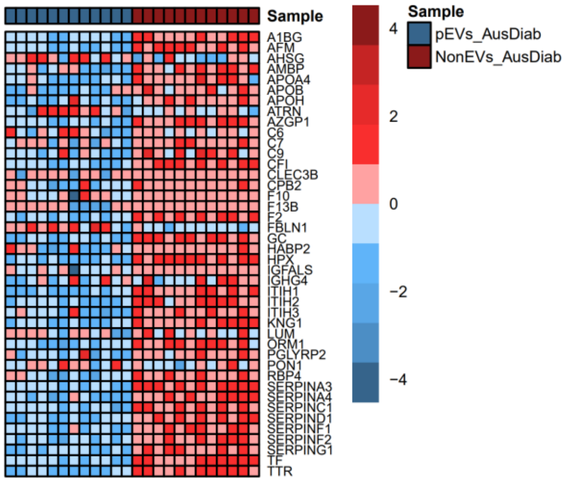


**Supplementary Figure 8. Heatmap of pEV and NonEV protein features in AusDiab validation set.**

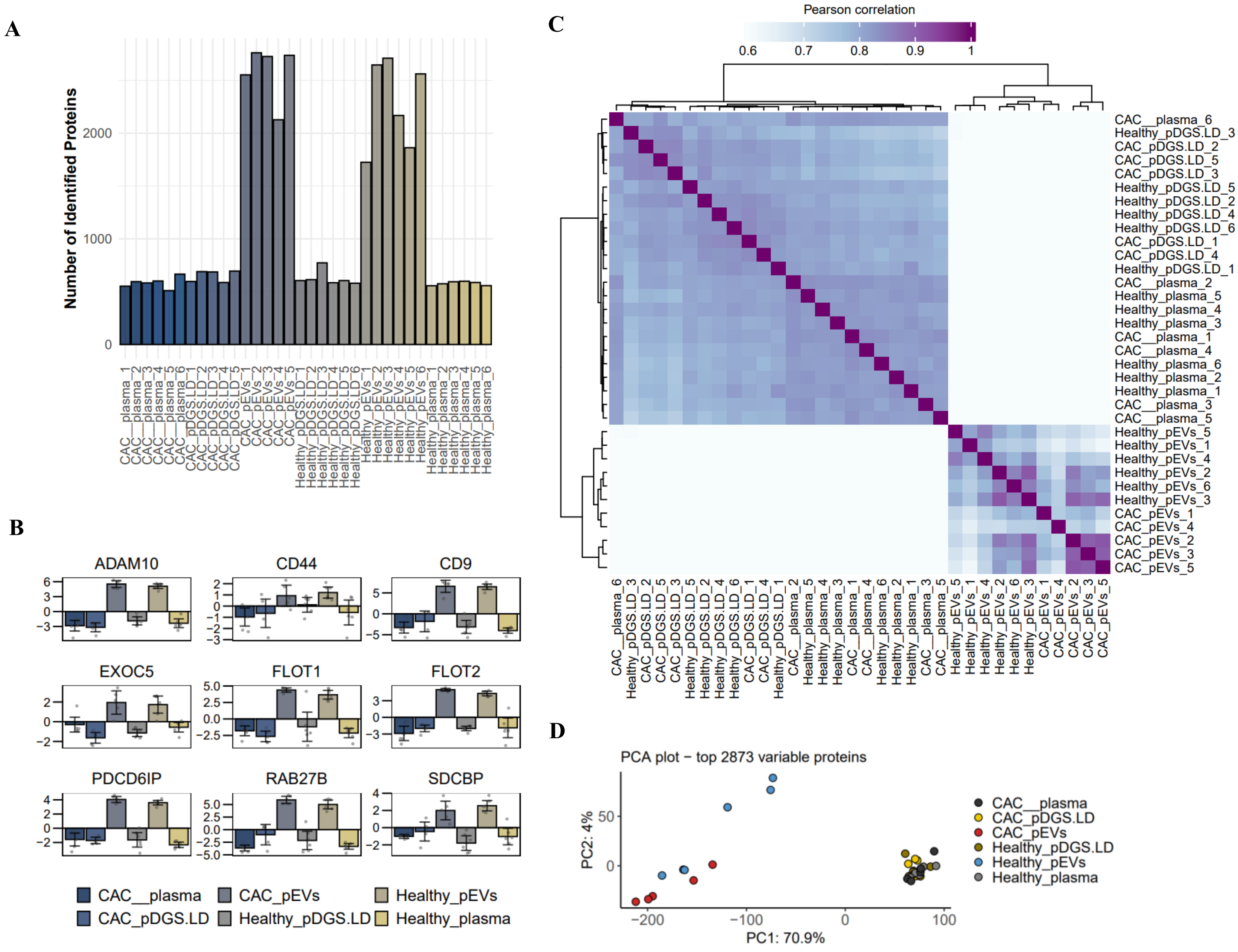


**Supplementary Figure 9. Proteome analysis of pEVs, NonEVs and neat plasma from EDCAD set.** Individuals with either positive CAC score (CAC group) or zero CAC score (Healthy group) are indicated. **A.** Proteins quantified in pEVs, NonEVs and neat plasma from individuals with either positive CAC score (CAC group) or zero CAC score (Healthy group). **B.** Bar plot depicting normalised intensities (Z-scored) of indicated proteins. **C.** Heatmap of Pearson correlation of quantified proteins. **D.** Principal component analysis of quantified proteins; error bars denote the standard error of the mean.

**A**

**B**

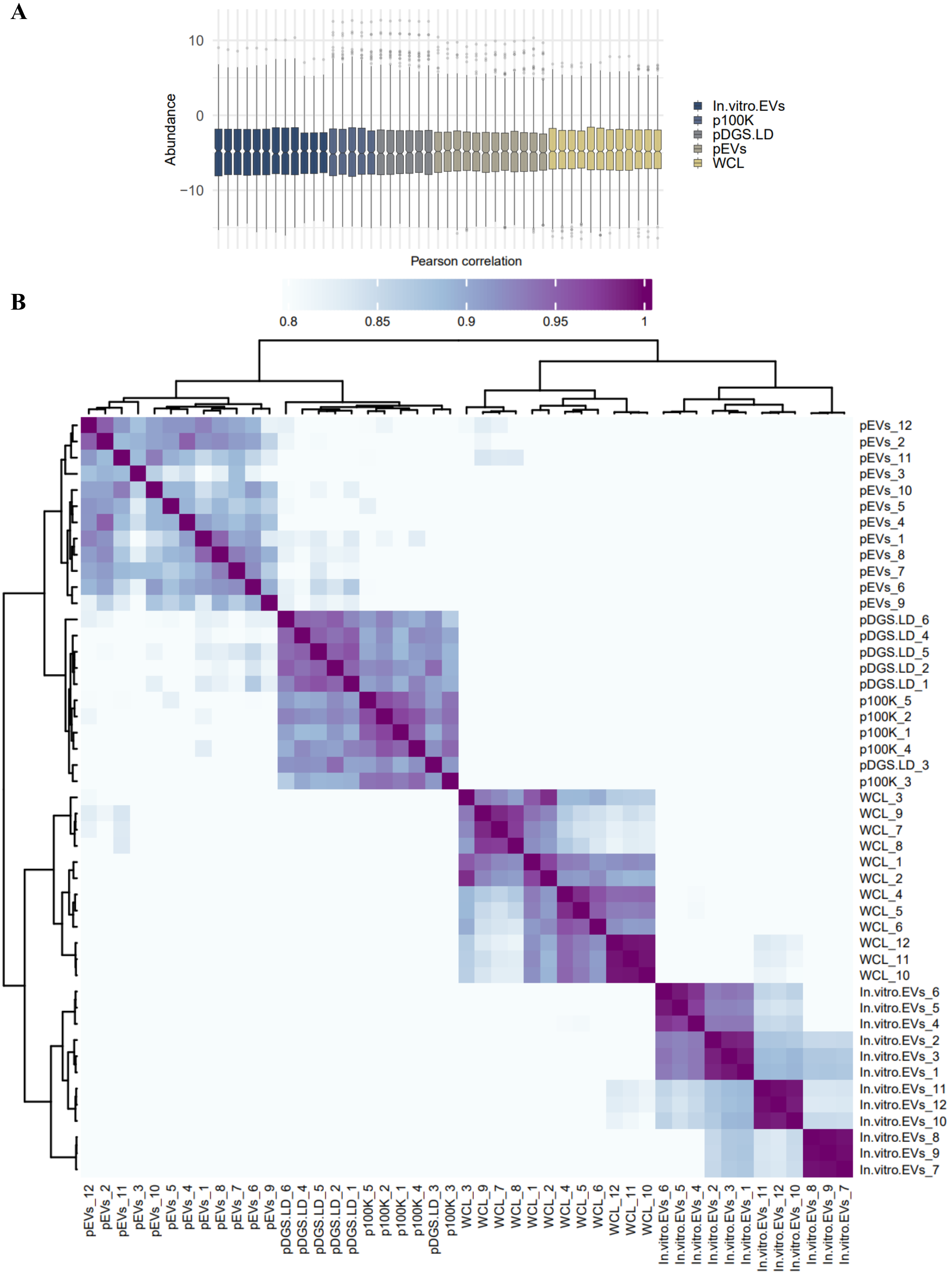


**Supplementary Figure 10. Lipidome profiling of circulating EVs. A.** Lipid abundance (MS-based intensity) for in vitro EVs, pEVs, p100K, pDGS.LD, and cells (WCL). Boxplots show the median (centre line), 25th–75th percentiles (box), minima and maxima within 1.5× interquartile range (whiskers), and outliers beyond. **B.** Heatmap of Pearson correlation matrix of lipid abundance between datasets.

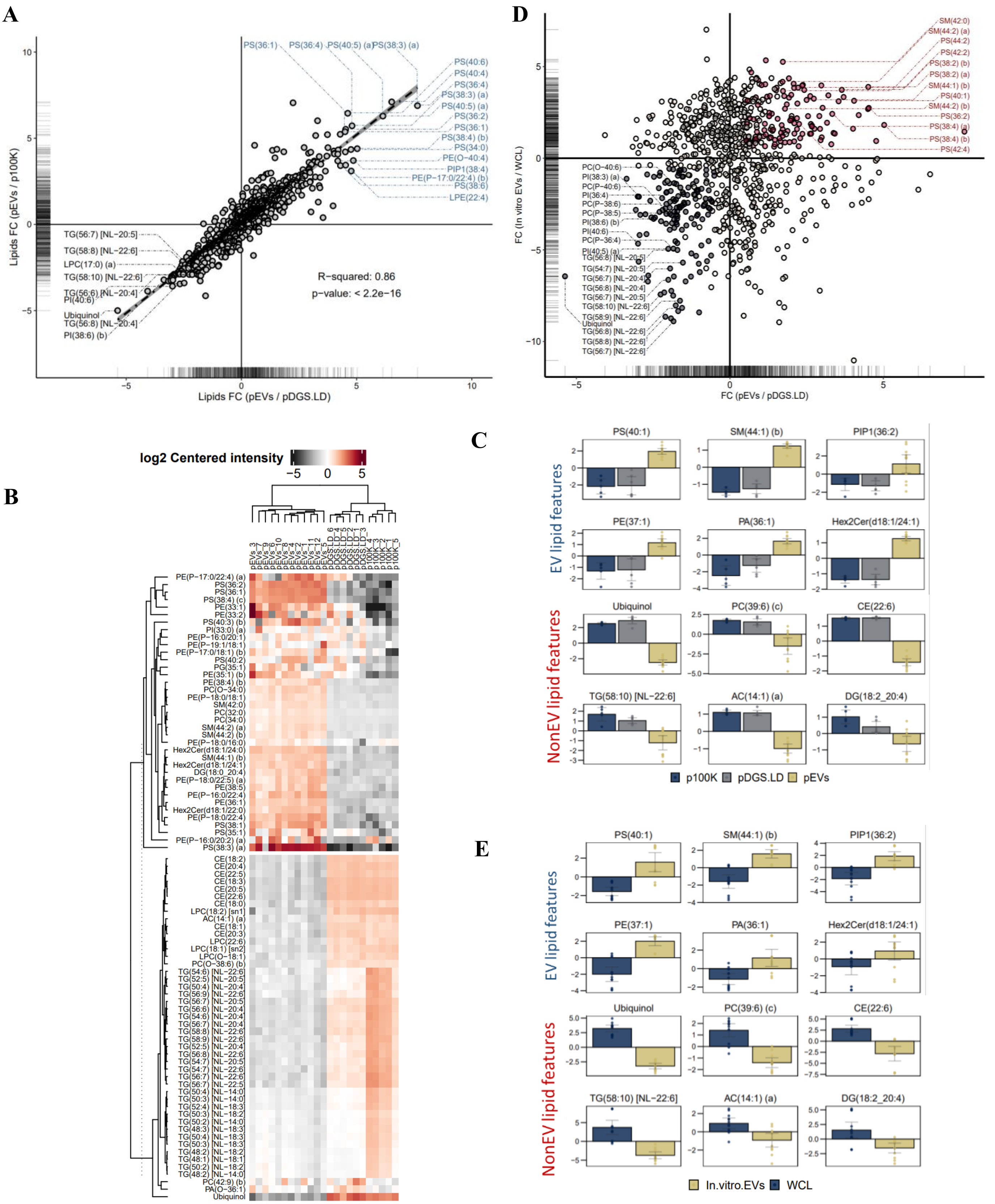


**Supplementary Figure 11. Lipid species co-enriched in circulating EVs and EVs from cell culture. A.** Scatter plot showing relative abundance of lipids in pEVs vs p100K and pEVs vs pDGS.LD. Lipids in blue are enriched in pEVs. **B.** Heatmap depicting differentially abundant lipids in pEVs vs NonEVs. **C.** Scatter plot showing relative abundance of lipids in pEVs vs p100K/pDGS.LD and in vitro EVs vs WCL lipidome data sets. Blue circles (lipid markers) represent lipids with significantly greater abundance (Log2 FC > 0.5 & p value < 0.05) in pEVs vs pDGS.LD and in vitro EVs vs cells. Red circles (exclusion lipids) represent lipids with significantly lower abundance (Log2 FC < -0.5 & p value < 0.05) in pEVs vs pDGS.LD and in vitro EVs vs cells. Grey circles represent lipids that do not meet the above criteria. **D-E.** Bar plots showing relative abundance (log2 centred intensities) of differentially abundant (p<0.05) lipids in indicated lipidomes; error bars denote the standard error of the mean..

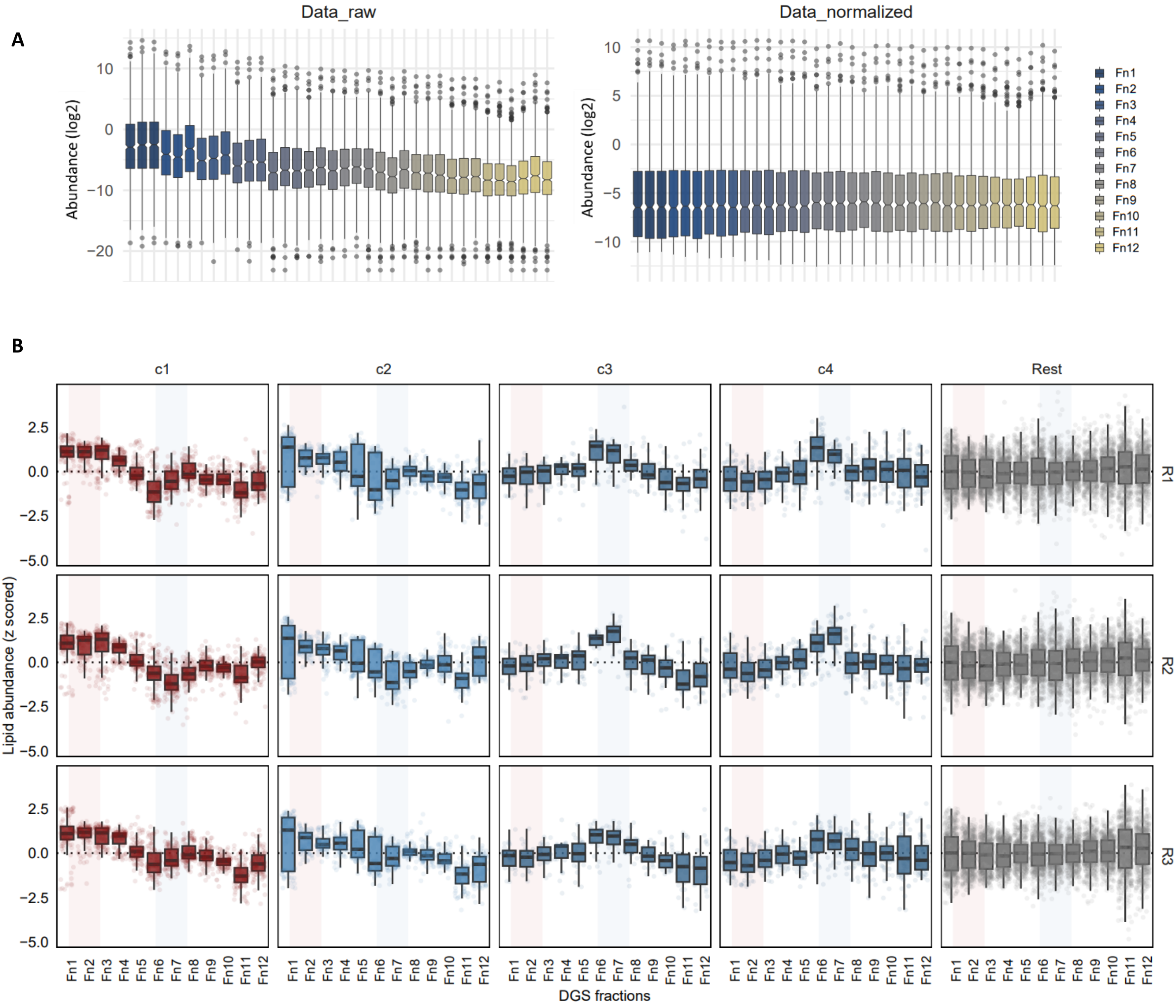


**Supplementary Figure 12. PS lipid class is enriched in EVs in plasma. A** Lipid abundance (normalized) across 12 fractions of density gradient separated plasma. Boxplots show the median (centre line), 25th–75th percentiles (box), minima and maxima within 1.5× interquartile range (whiskers), and outliers beyond. **B.** Boxplots depict abundance of cluster 1-4 lipids for DGS fractions for each biological replicate (R1-3). Y axis represents Z-scored abundance (MS-based abundance for each lipid - mean abundance) / standard deviation (Z score normalization). Blue lines highlights fractions 6-7 (corresponding to pEV fractions), whereas red line depicts fractions 1-3 (corresponding to pDGS.LD fractions). Boxplot show the median (centre line), 25th–75th percentiles (box), minima and maxima within 1.5× interquartile range (whiskers).

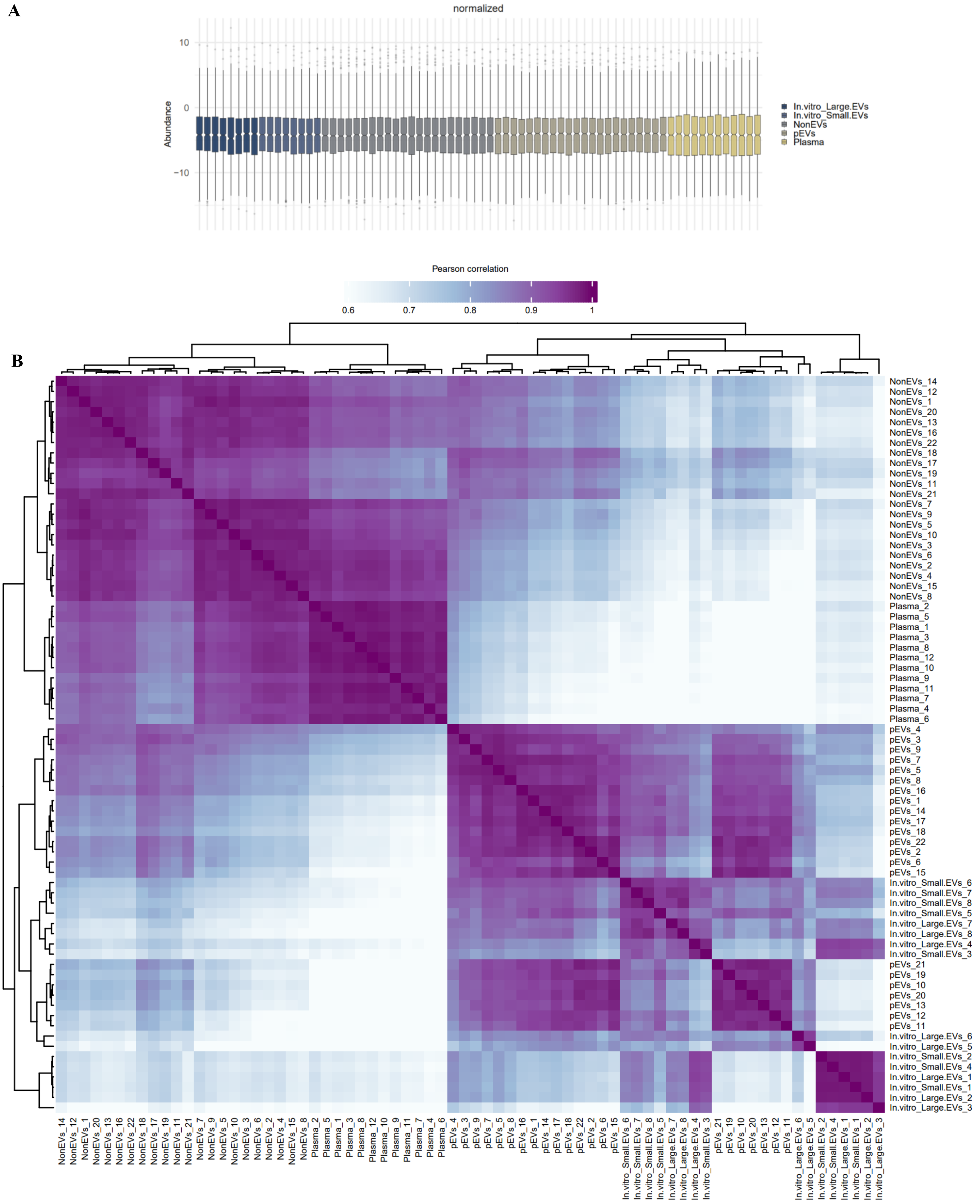


**Supplementary Figure 13. Lipidome analysis of pEVs from AusDiab validation cohort and non-transformed cells**. **A.** Boxplot depicting normalised abundance (vsn normalized) of lipids quantified in non-transformed EVs (in vitro Small EVs or in vitro Large EVs) or pEVs, NonEVs or neat plasma from AusDiab validation set. Boxplots show the median (centre line), 25th–75th percentiles (box), minima and maxima within 1.5× interquartile range (whiskers), and outliers beyond. **B.** Heatmap of Pearson correlation of quantified lipids.

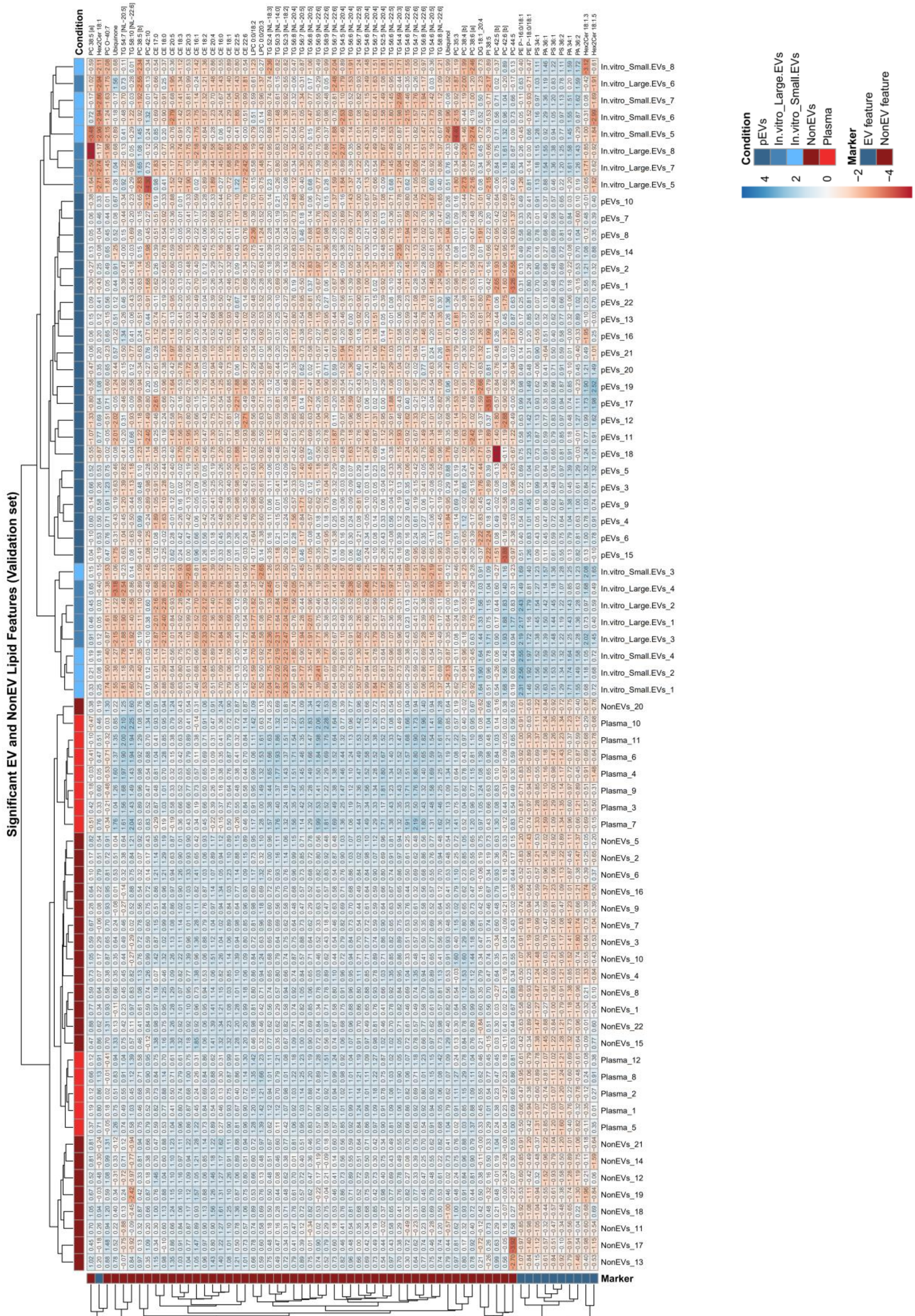


**Supplementary Figure 14.** Heatmap of lipid features distribution in pEVs versus NonEVs from AusDiab validation cohort and EVs from non-transformed cells

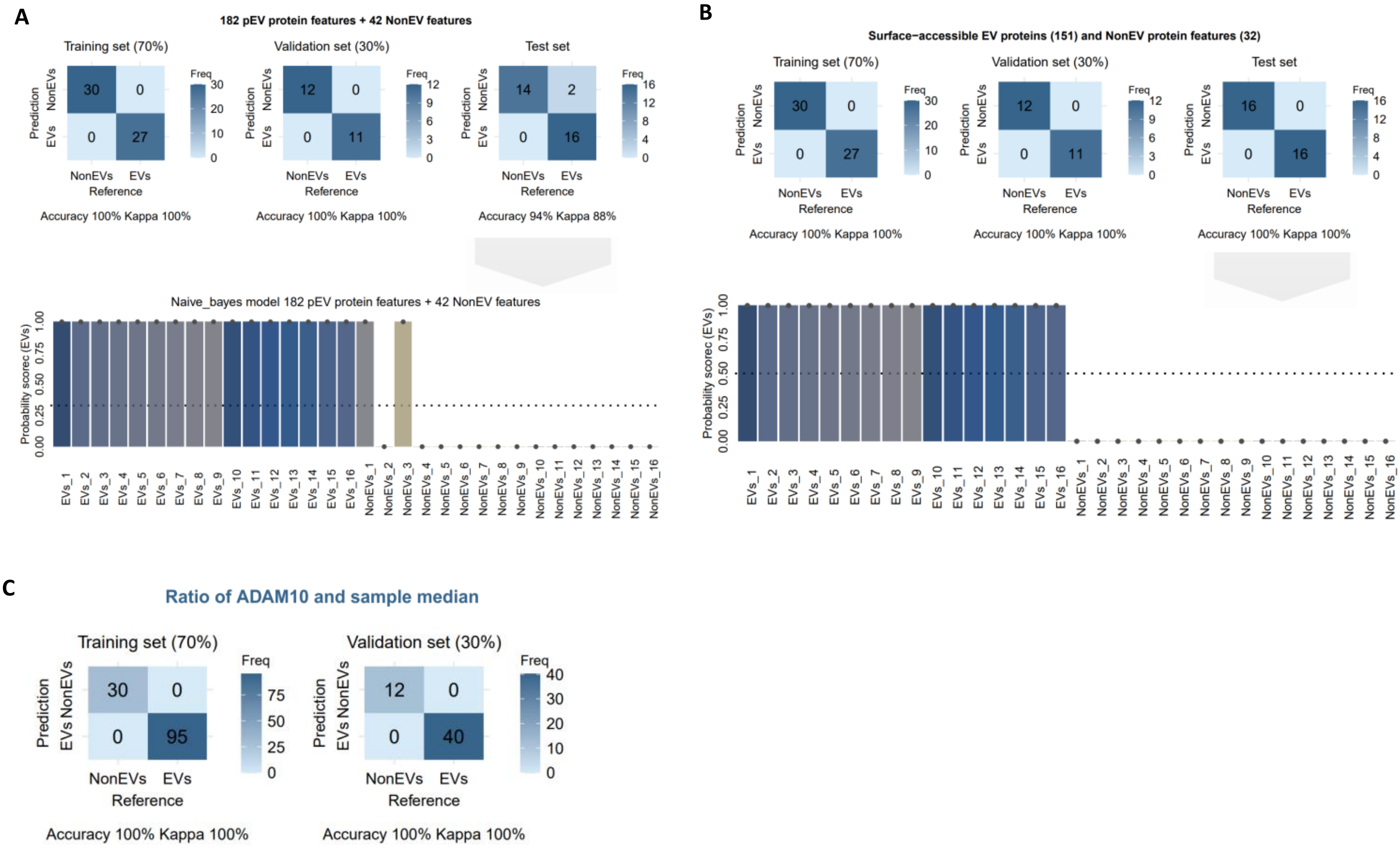


**Supplementary Figure 15. Machine learning classification of EV and NonEV particles in plasma using protein features. A.** Confusion matrix (using the ensemble model) of training set (70%), remaining validation set (30%), and independent test set, for cluster 1 proteins, which include top 97 proteins enriched in EVs (FC>5) and 28 exclusion proteins. Probability scores for sample classification into EVs using ensemble model on independent test set. Greater scores indicate higher confidence in predicting sample belonging to EV class. **B.** Confusion matrix using nnet classifier of validation set (30%) and independent test set, for 126 pEV surface proteins. Bar plot show probability scores for sample classification into EVs using nnet model on independent test set. **C.** Confusion matrix using nnet classifier of validation set (30%) and independent test set, for ADAM10 vs sample median intensities for each proteome as depicted in Figure 5B.

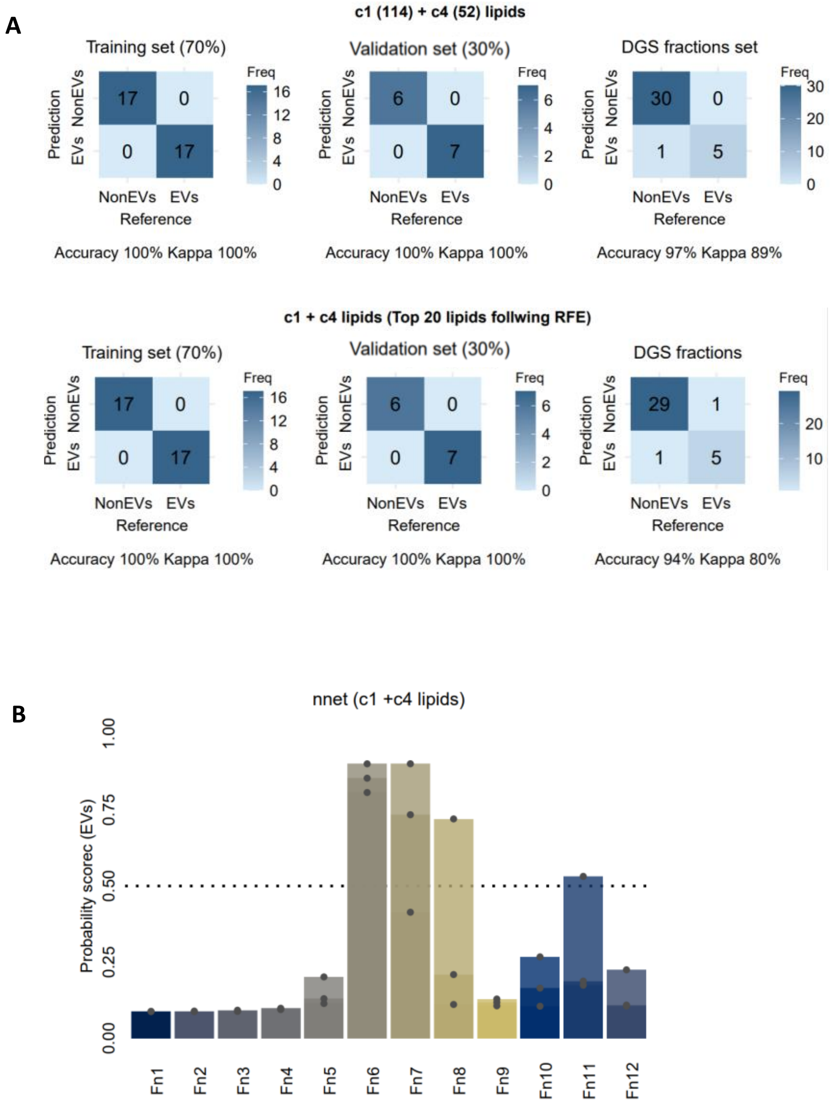


**Supplementary Figure 16. Machine learning classification of EV and NonEV particles in plasma using lipid features. A** Confusion matrix using Neural Network algorithm ('nnet') classifier of training set (70%), validation set (30%) and independent test set, for cluster c1 and c4 lipids (upper panel), and top 20 c1 and c4 lipid features following reclusive feature elimination (lower panel). **B.** Bar plot indicated probability scores for DGS fraction lipidomes classification into EVs using 'nnet' model based on top 20 c1 and c4 lipid features on independent test set. Greater scores indicate higher confidence in predicting DGS fractions belonging to EV class.

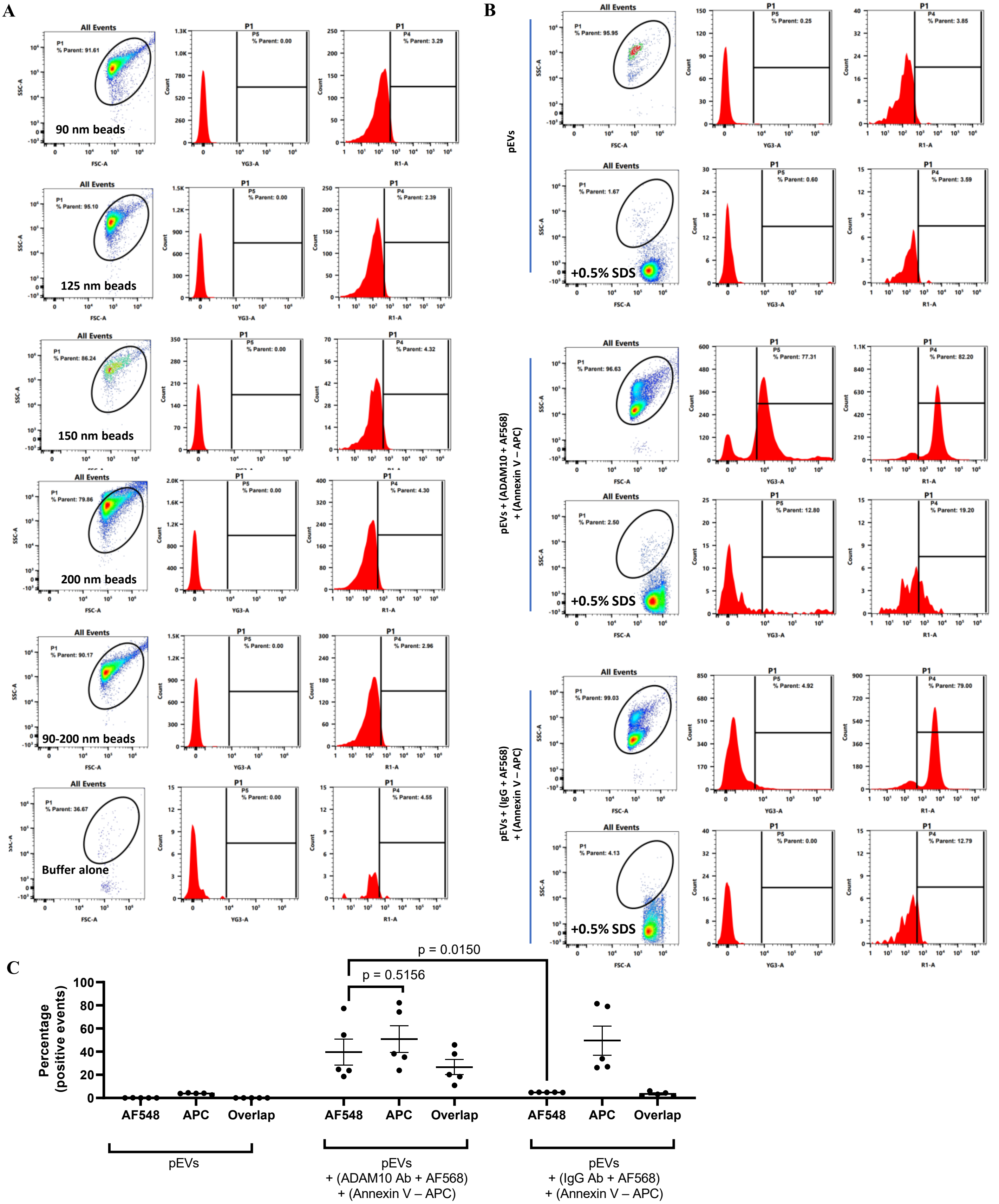


**Supplementary Figure 17. Spectral flow cytometry (Cytek Aurora)-based pEV analysis for ADAM10 and PS expression A.** Scatter plot depicts instrument gating calibration using 90 nm, 125 nm, 150 nm, 200 nm and equal mix (90-200 nm) beads. Bottom panel indicated buffer alone control. YG3-A channel used for detecting Alex Fluor 568 signal. R1-A channel used for detecting APC signal. **B.** Cytek Aurora-based pEV analysis. Scatter plot showing detection of ADAM10 antibody-AF568 positive (detected using YG3-A channel) and PS positive (Annexin V-APC, detected using R1-A channel) in pEVs. IgG used as control for ADAM10 antibody. SDS detergent (0.5%) solubilization was performed to indicate EV origin of these signals. **C.** Plot depicting quantification of ADAM10+ (AF548 signal), PS+ (APC signal) or overlapping (ADAM10 and PS +) signal. Unpaired Two-tailed Student's t-test was performed. Data represents mean ± s.e.m., n=5 biological replicates per group.

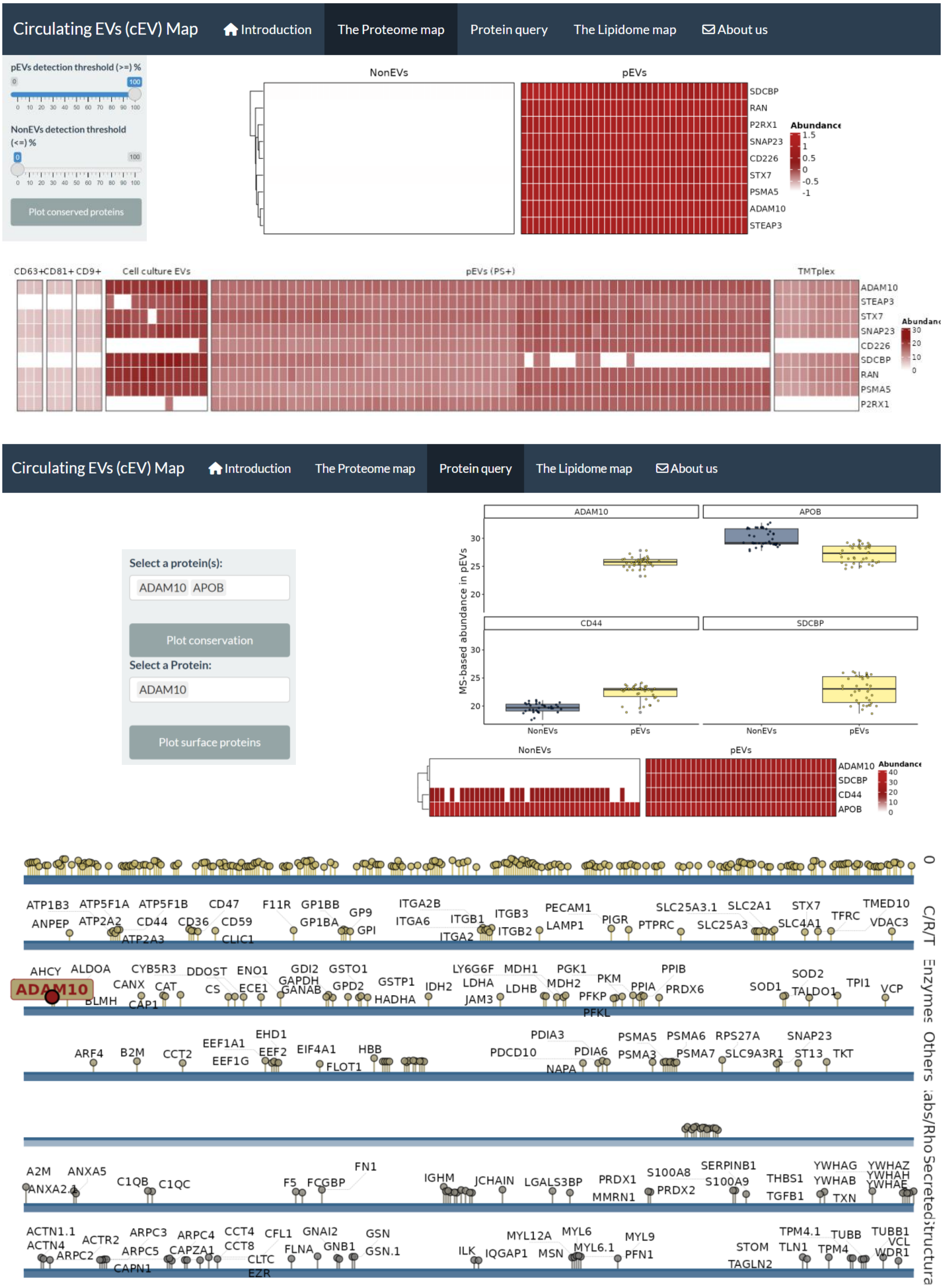


**Supplementary Figure 18.** Interrogating protein of interest expression in circulating EVs using the Shiny app.

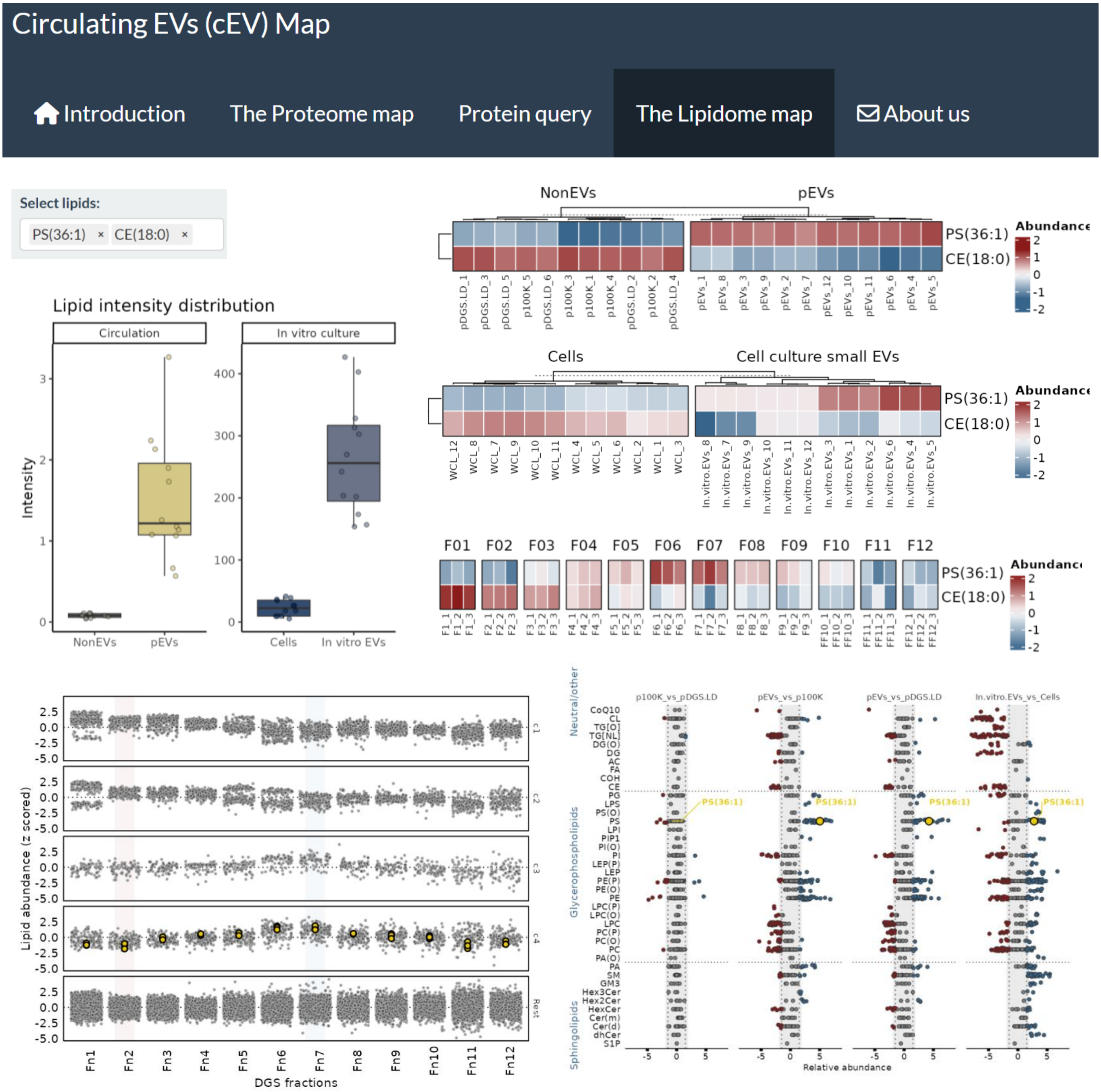


**Supplementary Figure 19 .** Interrogating lipid of interest expression in circulating EVs using the Shiny app.

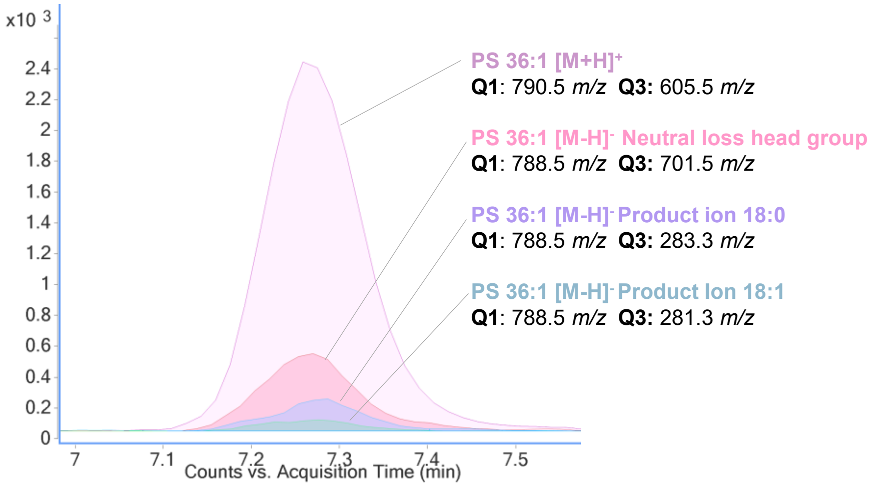


**Supplementary Figure 20. Characterization of PS 36:1 in EVs using positive and negative ionization mode**. Pooled EV's were run under the same conditions in positive and negative ionization mode, looking for fragments corresponding to the neutral loss of the serine headgroup (red, positive mode, $[M+H]^+$) and all possible fatty acid species (negative mode, $[M-H]^-$), resulting in identification of 18:0 (283.3 *m/z*) and 18:1 (281.3 *m/z*).

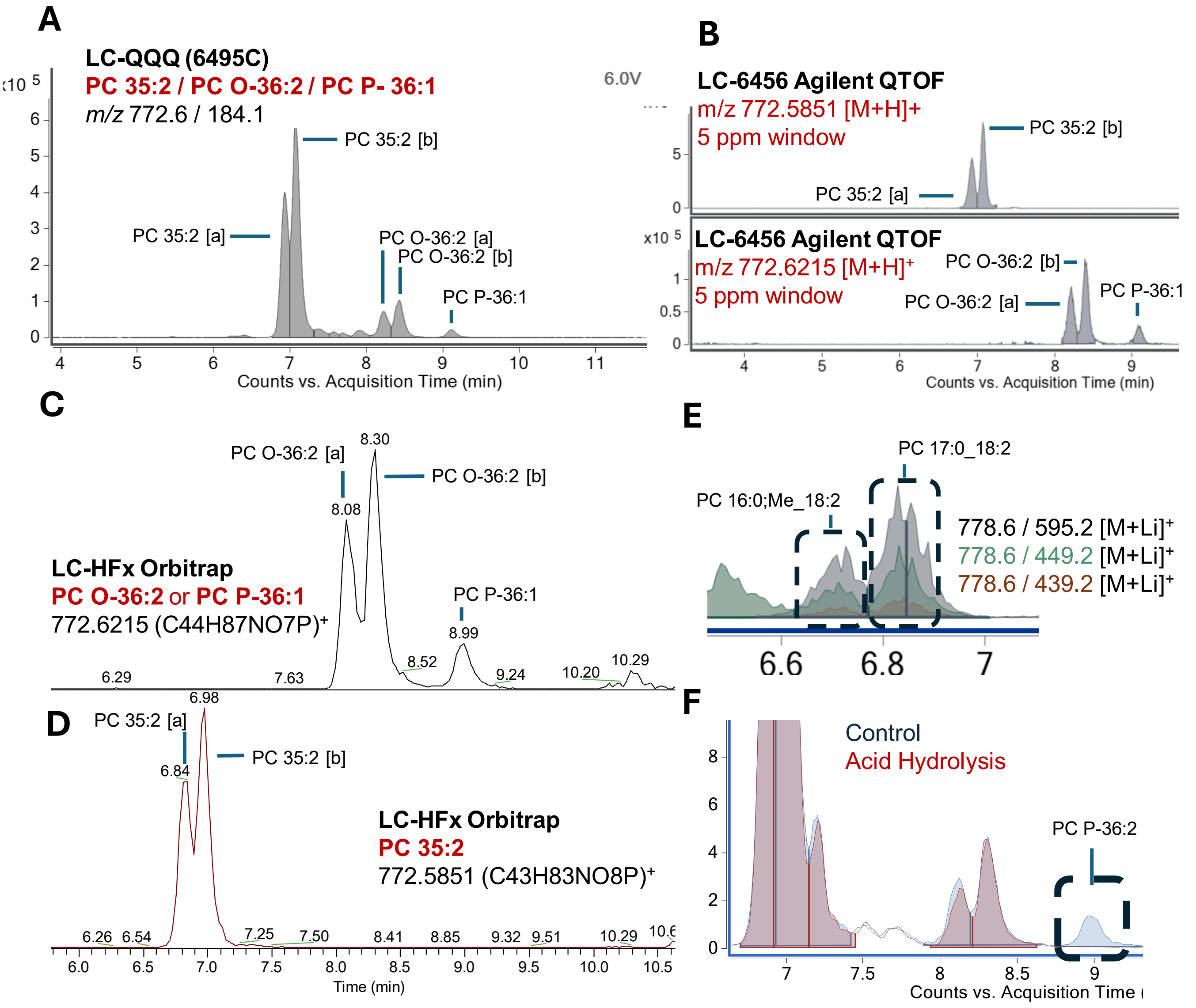


**Supplementary Figure 21. Validation of isobars and isomer separation between PC 35:2, PC O-36:2 and PC P-36:1 with chromatography. A -** Transition (772.6/184.1) corresponding to PC 35:2 / PC O-36:2 / PC P-36:1 using pooled human plasma (0.1µl on column injection) run on an Agilent 6495C with chromatographic conditions as previously described (Agilent Infinity II HPLC). **B -** EIC's corresponding to PC 35:2 and PC O-36:2 / PC P-36:1 using pooled human plasma (0.1µl on column injection) run on an Agilent 6546 QTOF with chromatographic conditions as previously described. Resolution is approximately 60,000 FWHM. The *m/z* 772.5851 (C43H83NO8P) putatively corresponds to PC 35:2 (Top panel) while 772.6215 (C44H87NO7P) corresponds to PC O-36:2 or PC P-36:1 (Panel). **C,D** - EIC's corresponding to PC 35:2 and PC O-36:2 / PC P-36:1 using pooled human plasma (0.1µl on column injection) run on an HFx Orbitrap and a Vanquish analytical HPLC with chromatographic conditions as previously described. Analysis was run as full MS1 scan with resolution set to 240,000 FWHM. The EIC 772.6215 (C44H87NO7P) corresponds to PC O-36:2 or PC P-36:1 (top panel) while EIC 772.5851 (C43H83NO8P) putatively corresponds to PC 35:2 (bottom panel). **E** - Lithium acetate was added to the running solvent. Species corresponding to PC 35:2 was measured as their lithium adduct $[M+Li]^+$ m/z 778.6, with the loss of the phosphocholine headgroup as the product ion (m/z 595.2). Product ions corresponding to the acyl composition 17:0 (449.2) and 18:2 was observed (439.2) split across two distinct chromatographic peaks. This was then followed up by subsequent synthesis of branched and straight isomer standards[134]. **F -** Transition (772.6/184.1) corresponding to PC 35:2 / PC O-36:2 / PC P-36:1 using pooled human plasma (0.1µl on column injection) run on an Agilent 6490 with chromatographic conditions as previously described. Red trace, HCl vapor treated, Blue trace, untreated control.

## Supplementary Figure 1A

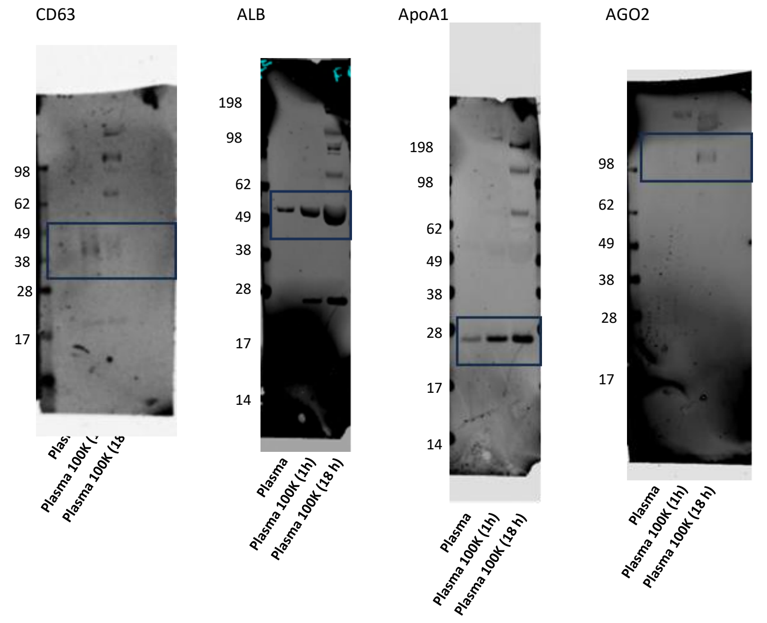


## Supplementary Figure 1C

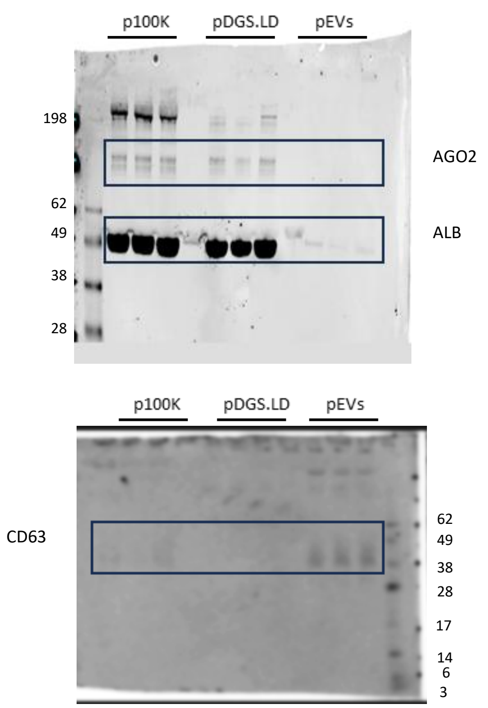


## Supplementary Figure 1D

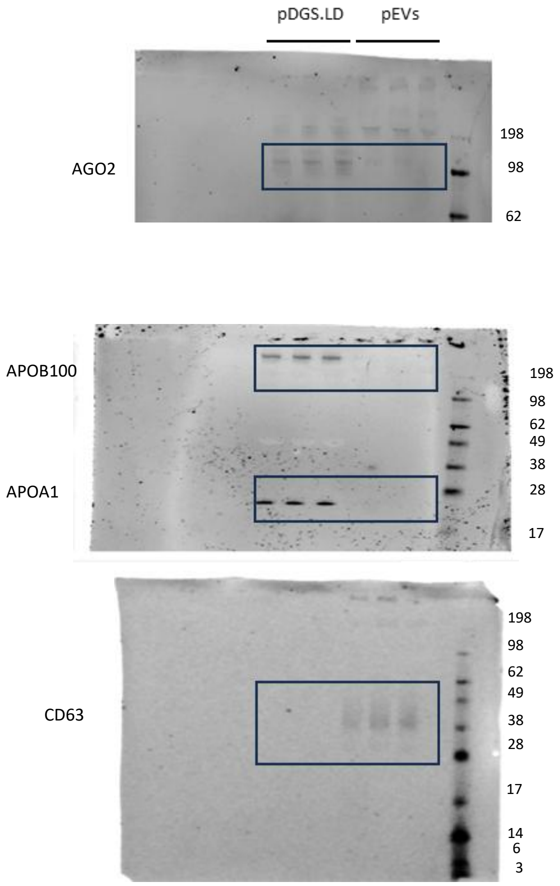


## Supplementary Figure 1H

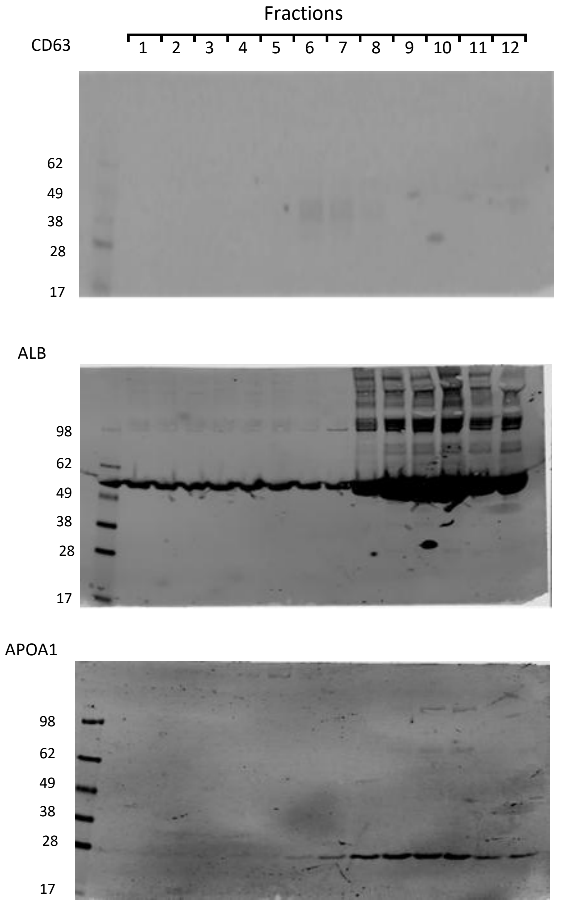


**Supplementary Figure 22. Uncropped Westerns**
