# Supplementary material for: Multi-omics identify hallmark protein and lipid features of small extracellular vesicles circulating in human plasma
Source: Nat Cell Biol. 2025 Nov 28;27(12):2167–85. doi: 10.1038/s41556-025-01795-7 (PMC12717007; doi:10.1038/s41556-025-01795-7)
Supplement: Supplementary file 2 — Reporting Summary [file 41556_2025_1795_MOESM2_ESM.pdf]

nature portfolio

Corresponding author(s): David Greening & Alin Rai

Last updated by author(s): Jul 19, 2025

Reporting Summary

Nature Portfolio wishes to improve the reproducibility of the work that we publish. This form provides structure for consistency and transparency in reporting. For further information on Nature Portfolio policies, see our Editorial Policies and the Editorial Policy Checklist.

Statistics

For all statistical analyses, confirm that the following items are present in the figure legend, table legend, main text, or Methods section.

n/a	Confirmed	
☐	☒	The exact sample size (n) for each experimental group/condition, given as a discrete number and unit of measurement
☐	☒	A statement on whether measurements were taken from distinct samples or whether the same sample was measured repeatedly
☐	☒	The statistical test(s) used AND whether they are one- or two-sided<br>*Only common tests should be described solely by name; describe more complex techniques in the Methods section.*
☒	☐	A description of all covariates tested
☐	☒	A description of any assumptions or corrections, such as tests of normality and adjustment for multiple comparisons
☐	☒	A full description of the statistical parameters including central tendency (e.g. means) or other basic estimates (e.g. regression coefficient) AND variation (e.g. standard deviation) or associated estimates of uncertainty (e.g. confidence intervals)
☒	☐	For null hypothesis testing, the test statistic (e.g. F, t, r) with confidence intervals, effect sizes, degrees of freedom and P value noted<br>*Give P values as exact values whenever suitable.*
☒	☐	For Bayesian analysis, information on the choice of priors and Markov chain Monte Carlo settings
☐	☒	For hierarchical and complex designs, identification of the appropriate level for tests and full reporting of outcomes
☐	☒	Estimates of effect sizes (e.g. Cohen's d, Pearson's r), indicating how they were calculated

Our web collection on statistics for biologists contains articles on many of the points above.

Software and code

Policy information about availability of computer code

Data collection	No software other than custom software or code was used for data collection. Software for data collection included Xcalibur software v4.0, MassHunter Acquisition v10, ZetaView (version 8.06.01 SP1), Cytek Aurora SpectroFlo (vS3.3.0)
Data analysis	No software other than custom software or code used for data collection. Software for data analysis included, GraphPad Prism software v.8.1.2/9.3.0, Image J (NIH Image Software), DIA-NN (v1.8). MaxQuant (v1.6.6.0-v1.6.14), MassHunter Quantitative Analysis 9.0, Cytek Aurora SpectroFlo (vS3.3.0), Microsoft Excel 2019 (Office365).

For manuscripts utilizing custom algorithms or software that are central to the research but not yet described in published literature, software must be made available to editors and reviewers. We strongly encourage code deposition in a community repository (e.g. GitHub). See the Nature Portfolio guidelines for submitting code & software for further information.

Data

Policy information about availability of data

All manuscripts must include a data availability statement. This statement should provide the following information, where applicable:

- Accession codes, unique identifiers, or web links for publicly available datasets
- A description of any restrictions on data availability
- For clinical datasets or third party data, please ensure that the statement adheres to our policy

Data generated or analyzed during this study are included in this published article (and its supplementary information files), source data or available from Data Repositories. Lipidomics data are available from the NIH Common Fund's National Metabolomics Data Repository (NMDR) website (in addition to quantiitittaive details in Supplementary Tables 31,40). Proteomics data are available from the ProteomeXchange Consortium. All MS-based proteomics data (including sample/label annotation) is deposited to the ProteomeXchange Consortium via the MASSive partner repository and available via MASSive with identifier (MSV000094307).

Hierarchical clustering was performed in Perseus using Euclidian distance and average linkage clustering, with missing values imputed at z-score 0. Proteome and lipidome data sets were analyzed using R package Differential Enrichment analysis of Proteomics data (DEP). Using DEP, the data was background corrected and normalized by variance stabilizing transformation (vsn, which also log2-transforms the data), followed by imputation of missing values, whereby missing at random data were 'knn' imputed and missing not at random data were 'MinProb' imputed. Protein-wise linear models combined with empirical Bayes statistics were used for the differential enrichment analysis, whereby the raw p-values were adjusted to correct for multiple testing using Benjamini-Hochberg method. Differentially abundant proteins or lipids were clustered by k-means clustering using DEP package. The PCA plot, Pearson Correlation matrix, volcano plots, log2 centred bar plots and overlap bar plots were also generated using DEP. Heatmaps were generated using ComplexHeatmap package (https://bioconductor.org/packages/release/bioc/html/ComplexHeatmap.html). Box plots and scatter plots were generated using RStudio package ggplot2 (https://rss.onlinelibrary.wiley.com/doi/abs/10.1111/j.1467-985X.2010.00676_9.x). Cytoscape was used to generate Ontology map (plugin v3.7.1). Bioconductor package clusterProfiler 4.023 was used to perform Ontology or KEGG pathway enrichment analysis, or gene set enrichment (KEGG) analysis, with default parameters used to identify significantly enriched gene sets. The pathway-based data integration and visualization was constructed using R package pathview. For identification of lipid-associated terms enriched in lipidomes, LION web-based ontology enrichment tool was used (www.lipidontology.com). For annotating surface proteins into different categories, SURFY-based categorical annotation of cell surface proteins were employed (https://wlab.ethz.ch/surfaceome/). We employed caretEnsemble R package to assess the ability of protein features (pEV protein features and NonEV protein features, using Naïve Bayes algorithm), surface protein features or lipid features, using Neural Network algorithm ('nnet')) to distinguish between distinguish between pEV and NonEV particles. The Shiny web application (https://evmap.shinyapps.io/evmap/) powered by R and hosted on shinyapps.io, was created using the R packages shiny, gplots, and ComplexHeatmap, and offers feature selections and visualizations for EV protein and lipid feature conservations in circulating EVs.

Field-specific reporting

Please select the one below that is the best fit for your research. If you are not sure, read the appropriate sections before making your selection.

☒ Life sciences ☐ Behavioural & social sciences ☐ Ecological, evolutionary & environmental sciences

For a reference copy of the document with all sections, see nature.com/documents/nr-reporting-summary-flat.pdf

Life sciences study design

All studies must disclose on these points even when the disclosure is negative.

Sample size	Proteomic experiment/file annotation is provided in ProteomeXchange Consortium via the MASSive partner repository and available via MASSive with identifier (MSV000094307) which details the number of biological replicates for each experimental condition (at least n=3). We have further included sample annotation for all proteomic and lipidomic data acquisition in this study in Source Data 3.
Data exclusions	All data exclusions are outlined in the methods, legends and/or Source Data/Supplementary information.
Replication	For all studies, groups and analyses contained at least three independent biological samples.
Randomization	For proteomic and lipidomic sample preparations, samples were blinded and later re annotated for data analysis.
Blinding	N/A

Reporting for specific materials, systems and methods

We require information from authors about some types of materials, experimental systems and methods used in many studies. Here, indicate whether each material, system or method listed is relevant to your study. If you are not sure if a list item applies to your research, read the appropriate section before selecting a response.

Materials & experimental systems

n/a	Involved in the study
☐	☒ Antibodies
☐	☒ Eukaryotic cell lines
☒	☐ Palaeontology and archaeology
☒	☐ Animals and other organisms
☐	☒ Human research participants
☒	☐ Clinical data
☒	☐ Dual use research of concern

Methods

n/a	Involved in the study
☒	☐ ChIP-seq
☐	☒ Flow cytometry
☒	☐ MRI-based neuroimaging

Antibodies

Antibodies used	Details of all antibodies are provided within the data supplement. Albumin (ab207327, Abcam), AGO2 (ab186733, Abcam) were used. Mouse antibodies CD63 (556019, BD Pharmingen), CD81 (555675, BD Pharmingen), APOB100 (3715-3-250, Mabtech), APOA1 (3710-3-1000, Mabtech) were used (1:1000). For flow, Anti-ADAM 10 Antibody (Sigma-Aldrich, AB19026; 2 µg in 100 ul vol), Rabbit IgG Isotype Control (Invitrogen, 10500C) were used. Secondary antibodies used were IRDye 800 goat anti-mouse IgG or IRDye 700 goat anti-rabbit IgG (1:15000, LI-COR Biosciences). For flow, Goat Anti-Rabbit Alexa Fluor 568 Dye secondary antibody (A-11011,

Thermo Fisher Scientific)

Validation	All antibodies used in this study have been validated by the manufacturer;<br>Albumin (ab207327, Abcam)<br>https://www.abcam.com/en-au/products/primary-antibodies/albumin-antibody-epr20195-ab207327<br><br>AGO2 (ab186733, Abcam)<br>https://www.abcam.com/en-au/products/primary-antibodies/argonaute-2-antibody-epr10411-ab186733<br><br>CD63 (556019, BD Pharmingen)<br>https://www.bdbiosciences.com/en-au/products/reagents/flow-cytometry-reagents/research-reagents/single-color-antibodies-ruo/purified-mouse-anti-human-cd63.556019<br><br>CD81 (555675, BD Pharmingen)<br>https://www.bdbiosciences.com/en-au/products/reagents/flow-cytometry-reagents/research-reagents/single-color-antibodies-ruo/purified-mouse-anti-human-cd81.555675<br><br>APOB100 (3715-3-250, Mabtech)<br>https://stella.mabtech.com/sites/default/files/product_datasheets/3715-3-250.pdf<br><br>APOA1 (3710-3-1000, Mabtech)<br>https://www.mabtech.com/api/files/product_datasheets/3710-3-1000.pdf<br><br>ADAM 10 (Sigma-Aldrich, AB19026)<br>https://www.sigmaaldrich.com/AU/en/product/mm/ab19026<br><br>Rabbit IgG Isotype (Invitrogen, 10500C)<br>https://www.thermofisher.com/antibody/product/Rabbit-IgG-Isotype-Control/10500C

Eukaryotic cell lines

Policy information about cell lines

Cell line source(s)	SW480 (CCL-288, ATCC), MDA MB 231 (HTB-26, ATCC), SW620 (CCL-227, ATCC) were from American Type Culture Collection (ATCC, VA, USA), and LIM1863 cells from Ludwig Institute for Cancer Research, Melbourne. Primary human cell source include human neonatal foreskin fibroblast cell line (neoHFF) [sourced as gift from P Kaur, Monash University, Australia), human dermal fibroblasts (hDF), adult (Gibco/Thermo Fisher Sci. #C0135C), human atrial cardiac fibroblasts (haCF) (Lonza, #CC-2903) and human ventricular cardiac fibroblasts (Lonza, #CC-2904), human umbilical vein endothelial cells (HUVEC, Lonza, #CC-2519; HUVEC sourced as gift [K Peter, BHDI, Australia], HUVEC-RFP, Angio-Proteomie #cAP-0001)
Authentication	Cells were grown in specified media and supplements as per detailed in methods (generation of cell conditioned media) and published protocols, and cells authenticated in our previous publications (PMID: 30582284, PMID: 23585443, PMID: 23230278, PMID: 38938901). Commercially purchased HUVEC, hDF, haCF and MDA MB 231 cells were not authenticated.
Mycoplasma contamination	Cells were routinely tested for mycoplasma (neg) contamination by PCR assay annually.
Commonly misidentified lines<br>(See ICLAC register)	This cell line is not registered on the list of misidentified cell lines.

Human research participants

Policy information about studies involving human research participants

Population characteristics	There was no population characteristics for Red Cross plasma samples, EDCAD cohort, or AusDiab cohort (other than donation age 18 years+).
Recruitment	Red Cross plasma samples were obtained blinded as pooled plasma samples, and plasma samples from individual donors already collected in the EDCAD cohort and AusDiab cohort study used in this study. There was no criteria for participant recruitment. Clinical parameters for EDCAD plasma samples are provided in Supplementary Table 41.
Ethics oversight	Human blood plasma samples were obtained from Australian Red Cross Lifeblood or EDCAD study. For Red Cross samples, ethical permits were obtained from the ethical committee of the Australian Red Cross Blood Service Human Research Ethics Committee, and La Trobe University Human Ethics Committee (HEC19485). For EDCAD samples, ethics permit was approved through Human Research Ethics Committee (HREC) at Baker Heart and Diabetes Institute and by the Alfred Hospital Ethics Committee (EDCAD-PMS, #492/20). For AusDiab trial, ethics permit was approved through HREC at Baker Heart and Diabetes Institute and by the Alfred Hospital Ethics Committee (#39/11).

Note that full information on the approval of the study protocol must also be provided in the manuscript.

Flow Cytometry

Plots

Confirm that:

☒ The axis labels state the marker and fluorochrome used (e.g. CD4-FITC).

☒ The axis scales are clearly visible. Include numbers along axes only for bottom left plot of group (a 'group' is an analysis of identical markers).

☒ All plots are contour plots with outliers or pseudocolor plots.

☒ A numerical value for number of cells or percentage (with statistics) is provided.

Methodology

Sample preparation	Plasma EVs (~5 µg) were subjected to fixation and permeabilization using eBioscience™ Foxp3 / Transcription Factor Staining Buffer Set (Invitrogen™, 00-5523-00). Briefly, pEVs (in 50 µl PBS) was incubated with 500 µl fixation and permeabilization buffer on ice for 30 mins. Samples were ultracentrifugation at 100,000 g (1 h at 4 °C) and pellets resuspended in 100 µl wash buffer. Samples were stained with 5 µl APC Annexin V reagent (BioLegend) and either 2 µg of Anti-ADAM 10 Antibody (Sigma-Aldrich, AB19026) or Rabbit IgG Isotype Control (Invitrogen, 10500C). Samples were incubated at room temperature (gentle end-over mixing) for 1h. Samples were topped with 900 µl wash buffer and washed twice (ultracentrifuged at 100,000 g (1 h at 4 °C)) to remove any remaining antibodies and potential antibody aggregates. The pellets resuspended in 100 µl wash buffer containing 0.5 µl of Goat Anti-Rabbit Alexa Fluor 568 Dye secondary antibody (ThermoFisher Scientific) incubated for 30 mins at room temperature (gentle end-over mixing) in dark. Samples were topped with 900 µl wash buffer and washed twice (ultracentrifuged at 100,000 g (1 h at 4 °C)). Pellets resuspended in 100 µl of PBS (0.5% bovine serum albumin) filtered using 0.22 µm filter
Instrument	Cytek Aurora flow cytometer
Software	SpectroFlo (vS3.3.0)
Cell population abundance	N/A
Gating strategy	Instrument gating calibration was performed using 90 nm (#64009-15) 125 nm (#64011-15), 150 nm (#64012-15), 200 nm (#64013-15) and equal mix (90-200 nm) beads (Nanobead NIST Traceable Particle Size Standards). The threshold for side scatter was set to 430, and the gain of side scatter (SSC) were set to 10. YG3-A channel used for detecting Alex Flour 568 signal, R1-A channel used for detecting APC signal and 10 000 events were recorded for all samples with the slowest flow rate to minimize the swarming effect.

☒ Tick this box to confirm that a figure exemplifying the gating strategy is provided in the Supplementary Information.
