# Supplementary figures and images for: Multi-omics identify hallmark protein and lipid features of small extracellular vesicles circulating in human plasma
Source: Nat Cell Biol. 2025 Nov 28;27(12):2167–85. doi: 10.1038/s41556-025-01795-7 (PMC12717007; doi:10.1038/s41556-025-01795-7)

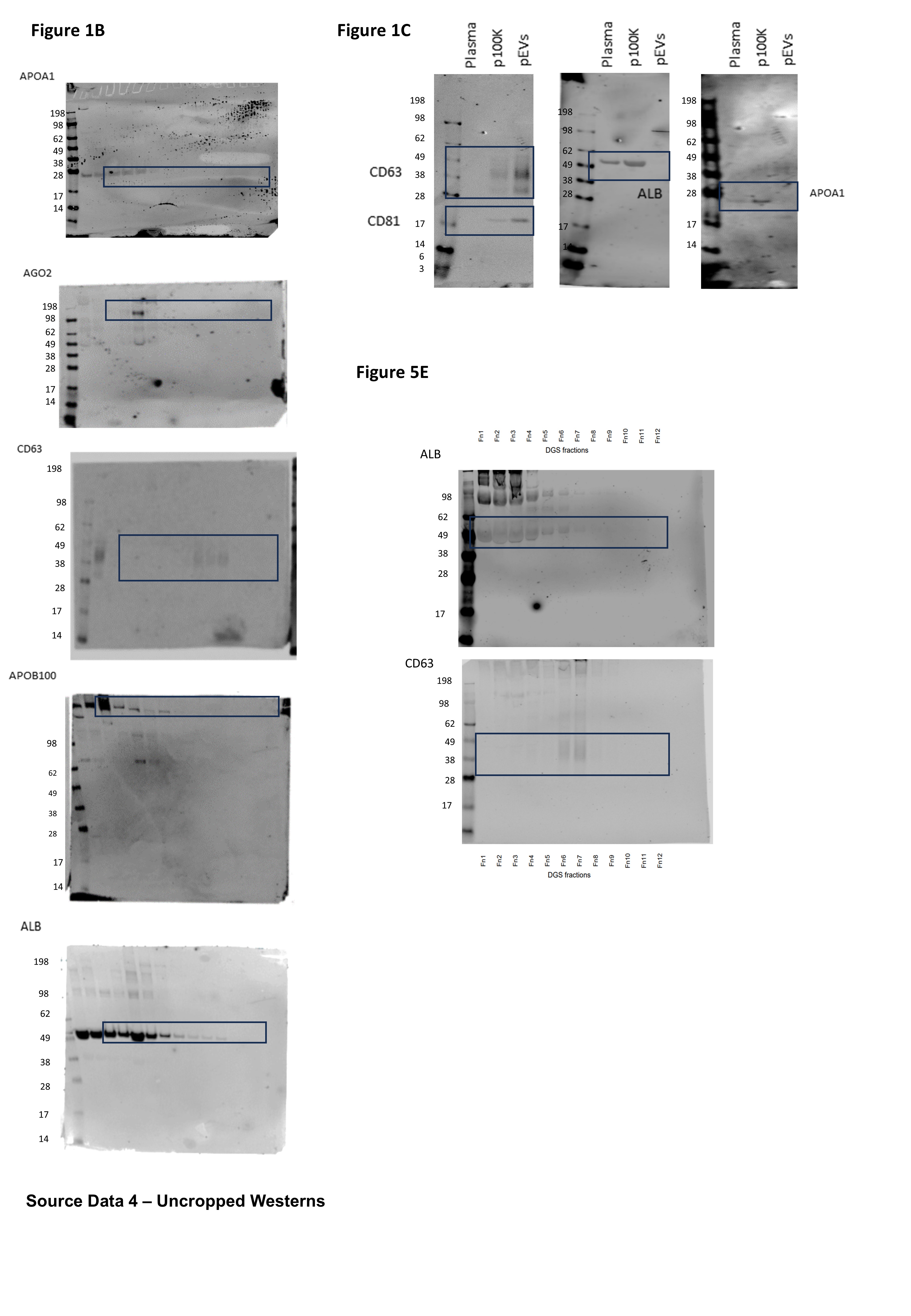

Supplement: Supplementary file 6 — Source 4; uncropped western blots. [file 41556_2025_1795_MOESM6_ESM.jpg]
